# Thermo solutal convective transport of aqueous Fe_3_O_4_ nanofluid in an inclined porous annulus under combined thermophoretic and electrophoretic forces

**DOI:** 10.1038/s41598-025-27022-9

**Published:** 2025-12-02

**Authors:** B. Shilpa, Irfan Anjum Badruddin, R. Gangadhara Reddy, Sarfaraz Kamangar, Mohammad Zuber, Essam R. I. Mahmoud

**Affiliations:** 1 Department of Mathematics, Dayananda Sagar College of Engineering, Bangalore, 560078 India; 2 Mechanical Engineering Department, College of Engineering, King Khalid University, 61421 Abha, Saudi Arabia; 3 Department of Mathematics, AMC Engineering College, VTU, Bangalore, India; 4 Department of Aeronautical & Automobile Engineering, Manipal Institute of Technology, Manipal Academy of Higher Education, Manipal, 576104 India; 5 Department of Mechanical Engineering, Islamic University of Madinah, 41411 Madinah, Saudi Arabia

**Keywords:** MHD, Electrophoretic and thermophoretic particle deposition, Viscous dissipation, Double-diffusive convection, Thermal radiation, Finite difference method, Engineering, Mathematics and computing, Nanoscience and technology, Physics

## Abstract

This computational study examines the electrophoretic and thermophoretic particle deposition, as well as the mass and heat transfer, of a dissipative and radiative Fe_3_O_4_ aqueous nanofluid flow in a porous, inclined annular medium under the influence of a magnetic field. The modelling of the current problem resulted in a complex, non-linear system of partial differential equations. These equations are solved by adopting a finite difference approach. The computed results are in exceptional agreement with those of existing results. This study demonstrates that for the normal incidence of inclination $\left( {0 \le \chi \le 30^{0} } \right)$, hydrostatic pressure plays a major role in the axial velocity and also when the annulus is horizontal maximum axial velocity moves downward. Adjusting the radiation parameter improves thermal efficiency and homogeneity in nanofluid-based heating systems. As the values of the electrophoretic particle deposition parameter increase, the concentration of the nanofluid enhances, whereas the concentration diminishes with a rise in the thermophoretic particle deposition parameter. Higher values of radiation parameters increase the nanofluid’s heat transfer rate and the increased values of the viscous dissipation diminish the heat transfer rate.

## Introduction

The research on heat and flow in porous annular regions offers substantial benefits by enhancing thermal efficiency, improving fluid management, and providing insights into complex flow behaviors, making it a critical area of research in engineering and environmental sciences. Recently, Nield and Simmons^1^ presented a mathematical representation to show the heat transport behaviour in a porous medium. In their study of Rogers–Horton-Lapwood issue with significant anisotropy and heterogeneity, Nield and Kuznetsov^2^ looked at a straightforward scenario in which two isotropic and homogeneous layers in a horizontal plane produced heterogeneity. The authors first developed and statistically computed the eigenvalue issue by deriving a novel hydrodynamic boundary value at the point of interaction between two different porous media. Nield and Bejan^3^ have presented a chapter to express the first law of thermodynamics in a permeable region. The authors have considered an isotropic medium with negligible radiation, heat dissipation, and work done through pressure variations. The effects of solid boundaries and momentum forces on mass transport in a permeable medium have been investigated numerically and experimentally by Vafai and Tien^4^. The authors used the local volume-averaging approach to solve the model while considering the mass transport via the permeable medium close to an impermeable border. Heat transmission and liquid flow in the interface zone created between two distinct porous media were analyzed by Vafai and Thiyagaraja^5^. The authors studied the interface area between a permeable and an impermeable region, as well as three kinds of interface regions between a liquid region and a permeable medium. Vafai and Tien^6^ explored the inertial forces and a solid border effect on heat transmission in a porous domain. The authors focused on analyzing the flow via a porous media near a non-permeable boundary. The TNE model and the internal heat production effect were considered by Rani et al.^7^ while analyzing the heat and fluid flow via an internally heated porous layer. The authors estimated the field variables using the ANN model and the finite element approach (FEA). Leela et al.^8^ considered the impact of three different viscous dissipation models on heat transfer and addressed the problem using the FEA and ANN-based models.

Double-diffusive natural convection (DDNC) refers to natural convection that occurs when temperature and concentration gradients interact. The phenomena of DDNC in fluid-saturated porous material have been widely studied because of their significance in many applications. Examples include flow through packed beds, dispersion of chemicals in water-saturated soil, moisture migration in fiber insulation and grain storage, energy extraction from geothermal reservoirs, and chemical reactors used to separate or purify mixes.

DDNC fluxes in porous media have applications in geophysics, electrochemistry, metallurgy, and other domains. These characteristics have prompted theoretical, experimental, and numerical research on this phenomenon. Using the implicit finite difference approach, Hossain and Rees^9^ have examined the impact of buoyant forces on mass and heat diffusion via natural convective flow from an upward wavy surface. The authors concentrated on surface shear stress, heat transmission rate, and surface concentration gradient changes as a function of the governing variable. Shilpa and Leela^10^ have examined heat and solute transport in an inclined annulus, considering double diffusive convection, heat generation/absorption, and a distinct order of chemical reaction rates. The authors have addressed the coupled nonlinear system of PDEs by considering the implicit FDM approach. Using an implicit FDM, Hossain et al.^11^ evaluated the temperature-reliant viscosity influence on the free convection of a viscous fluid from a vertical undulating surface. Shilpa et al.^12^ examined the DDC of a couple-stress fluid in porous channels considering the linearly varying wall temperature boundary conditions. The authors have used the FEM to solve the modelled nonlinear coupled system of DEs of the model.

In recent years, the study of nanofluid magnetohydrodynamics (MHD) in permeable media has become a novel field of study. In such a situation, the imposed magnetic field reduces flow velocities and degrades heat exchange, but the high surface area of the porous medium and the suitable thermophysical characteristics of the nanofluid tend to increase heat transmission. Here are a few recent works on the topic. Khaled Al-Farhany et al.^13^ have investigated the quantitative dynamics of a Fe3O4-aqueous fluid in an inclined curvilinear lid-driven space under the influence of an angled magnetic field. The authors demonstrated that while the average *Nu* and *Sh* drop with rising Hartmann numbers, they rise with enhancing *Re*, nanoparticle volume fraction, and fin length. The impact of a magnetic field and a distinct thermo-solutal source on stable DDC in a dual-sided cavity containing liquid potassium alloy has been investigated numerically by Gnanasekaran and Satheesh^14^ using the finite volume approach. According to the authors, the Reynolds number causes the heat and mass transmission to rise, whereas the Hartmann number causes them to fall. Using the Soret and Dufour effect in complex regions, Mohammadi and Gandjalikhan^15^ investigated the effect of radiation on DDC.

Numerous studies have focused on heat transmission analysis of nanofluids within enclosures. Due to density and temperature gradients, natural heat transfer takes place inside enclosures and is frequently employed in industrial and technological settings. This phenomenon affects thermal insulating systems in buildings, solar collectors, commercial heat exchangers, fiber insulation, nuclear cooling elements, grain storage regions, and room air conditioners. Due to its significance, recent numerical and experimental research has focused on free convection. Fe_3_O_4_ nanoliquid packed in an enclosure between a rhombus and a wavy circular cylinder has been the subject of interest by Dogonchi and Hashim^16^ on free convection heat transport. Using a 2D nanofluid model, the authors have examined the effects of heat radiation and magnetic fields (MF) on three distinct forms of Fe_3_O_4_-water nanofluids. Also, Hosseinzadeh et al.^17^ experimentally reviewed the impact of a magnetic field on the friction factor and heat transfer enhancement of Fe_3_O_4_/water nanofluid. The calculations were done at various Reynolds number values and MF intensities in a device that contained a horizontal circular tube. The free convective flow and temperature efficiency characteristics of an inclined, partially warmed rectangular permeable cavity filled with an electroconductive ternary nanofluid were examined by Thirumalaisamy et al.^18^ with viscous dissipation and a tilted magnetic field, and they used the Marker and Cell approach to obtain the solution. By taking non-uniform heat source/sink effects, Shilpa and Leela^19^ explored the HMT of three distinct nanofluids in a vertical annulus. The authors have determined that the Jeffrey fluid performs better than the Oldroyd-B and Maxwell fluids concerning heat transport. Suneetha et al.^20,21^ have analyzed the hybrid nanofluid flow HMT in a stretching sheet by considering irreversibility effect. Revathi et al.^22^ have explored the hybrid nanofluid flow and heat transmission in a microchannel considering activation energy and distinct heat sources. Also, Revathi et al.^23^ have investigated the nanofluid flow HMT in a porous medium considering thermal radiation and activation energy effect.

Thermal radiation plays a major role in many technical advances, including propulsion equipment for space vehicles, satellites, airplanes, nuclear power systems, and gas turbines. Consequently, several studies exist to illustrate the effects of radiative thermal transfer in nanofluids. In the boundary layer of a shifting magneto nanofluid, Sedki^24^ investigated the chemical processes, solar radiation, and Brownian movement effects on mixed convection HMT caused by a porous stretched surface that generates heat through a porous medium. Irfan et al.^25^ used a thermal non-equilibrium model to numerically assess heat transport by all three modes in a saturated permeable square cavity. The authors used the FEM to solve the governing PDEs, assuming that the flow complies with Darcy’s law.

As an energy source, viscous dissipation (VD) modifies temperature distributions and affects the heat transmission rate. The significance of VD is dependent on whether the plate is frozen or warmed. Exact examples of real-world applications where the final output of desired features depends on the freezing pace and stretching process include the ejection of heat towards the creation of materials, the production of paper, the freezing of electronic chips, and so on. VD is a prime instance of this phenomenon. Fand and Buckner^26^ and Fand et al.^27^ investigated the VD influences on free convection in a flat cylinder immersed in porous media. Their analysis indicated that VD should not be overlooked. Saeid and Pop^28^ examined the effect of VD on free convection in a permeable cavity and noticed that increasing the VD parameter affects the heat transport in the hot region. Israel-Cookey et al.^29^ evaluated the impact of VD and radiative unsteady MHD convective flow across a permeable vertical plate. They found that increasing VD results in an augmentation in the thermal profile. Leela et al.^8^ explored the effect of three distinct VD models on heat with flow in a microporous channel by considering the induced magnetic field effect. Irfan et al.^30^ analyzed heat transport with the impact of radiation and VD in a square porous cavity. The authors presented the *Nu* at warm and cold walls of the cavity for distinct values of VD with radiation parameters. Also, Irfan et al.^31^ have explored the VD and radiation effect on convective flow in a permeable annular medium. The authors observed a reduction in the mean *Nu* at the hot surface and an enhancement in the mean *Nu* at the cold region for the increased values of the VD parameter.

According to a recent study, the mechanisms of gravity, convection, Brownian diffusion, electrophoresis, and thermophoresis are accountable for aerosol particle deposition. The distribution and rate of particle deposition on an annular surface will be examined in this work using thermophoresis and electrophoresis. A temperature gradient that pushes fluid from high to low temperatures and causes particles to travel from high to lower temperatures is known as the thermophoresis effect. Electrophoresis occurs when charged particles move and come in touch with one another. The electric field applied to particles, whether positively or negatively charged, affects their deposition rate differently. When particles are less than a micron, then the effects of electrophoresis and thermophoresis are substantial. As particle sizes increase, gravity and inertia become more important.

The connection between particle deposition and thermophoresis has been provided by many authors. The most often used connection was given by Tablot et al.^32^. The impact of thermophoresis on particle accumulation in laminar flow systems has been the subject of much research in the last few years. Batchelor and Shen^33^ discussed the effects of TPD in flow across flat surfaces, cylinders, and spinning objects using a similarity technique. Goren^34^ found that the thermophoretic energy is inclined to pull the particle away from the surface when the heat of the object’s surface is greater than the flow field. Tsai and Liang^35^ investigated the phenomena using thermophoresis when the region heat was lower than the fluid heat. In 2007, Postelnicu^36^ investigated the TPD effect on a horizontal permeable flat plate in a free convectional flow environment. In 2024, Shilpa et al.^37,38^ numerically examined the TPD effect on heat and solute transport of nanolubricants and tri hybrid nanofluids in stretching sheet and inclined annular geometry, considering radiation and non-linear heat source/sink effects. Particle deposition is influenced by thermophoresis, electrophoresis, and gravitational forces. Cooper et al.^39^ used convection–diffusion speed and electrophoretic velocity to estimate the deposition of particle rates in an axially symmetric and stagnant viscous flow model. A mathematical representation of the accumulation of particles in a 2D stationary flow field that takes electrophoresis, convection, diffusion, and gravity into consideration was presented by Turner et al.^40^. The governing equation was further developed using the similarity approach, which could be solved using the FDM, by Hwang and Daily^41^, who finished their analytical and empirical studies on silicon particle accumulation under the influence of an electric field in 1995. Tsai and Huang^42^ concentrated on the impact of electrophoretic and thermophoretic deposition in 2010. Chamkha and Pop^43^ have analysed the HMT past a vertical flat plate by considering the thermophoretic particle deposition effect. Theories of CCHF and generalised Fick’s relations were employed by Gangadhar et al.^44^ to examine the modern aspects of HMT. Salma et al.^45^ have explored the thermal conductivity variations of alumina nanofluid in an annulus.

The synergistic effects of electrophoretic and thermophoretic particle deposition, in conjunction with viscous dissipation and radiative heat transfer, on the thermo-hydrodynamic behavior of a Fe_3_O₄–water nanofluid in a porous inclined annular configuration are also investigated in this work. To the best of the author’s knowledge, no prior open literature report has included such a thorough investigation—integrating these concurrent transport mechanisms. The work meticulously clarifies the interaction between the previously described physical influences on heat and mass transport properties by using a robust finite difference approach. A deeper understanding of the underlying transport physics is provided by the graphical analysis of the temperature, concentration, and velocity fields, as well as the variation of Nusselt and Sherwood numbers.

Crucially, this study establishes the foundation for contemporary practical applications like the following while also enhancing the theoretical framework of nanofluid dynamics in porous structures:

- Magnetohydrodynamic energy systems and electrokinetic heat exchangers, where both electrophoretic and thermophoretic effects can be harnessed for enhanced thermal regulation.
- Biomedical cooling and targeted drug delivery devices, where understanding particle deposition under combined thermal and electric effects is vital.
- Microelectromechanical systems (MEMS) and microchannel thermal management, where precise control over heat and mass distribution is essential.

## Mathematical formulation

**Fig. 1:**
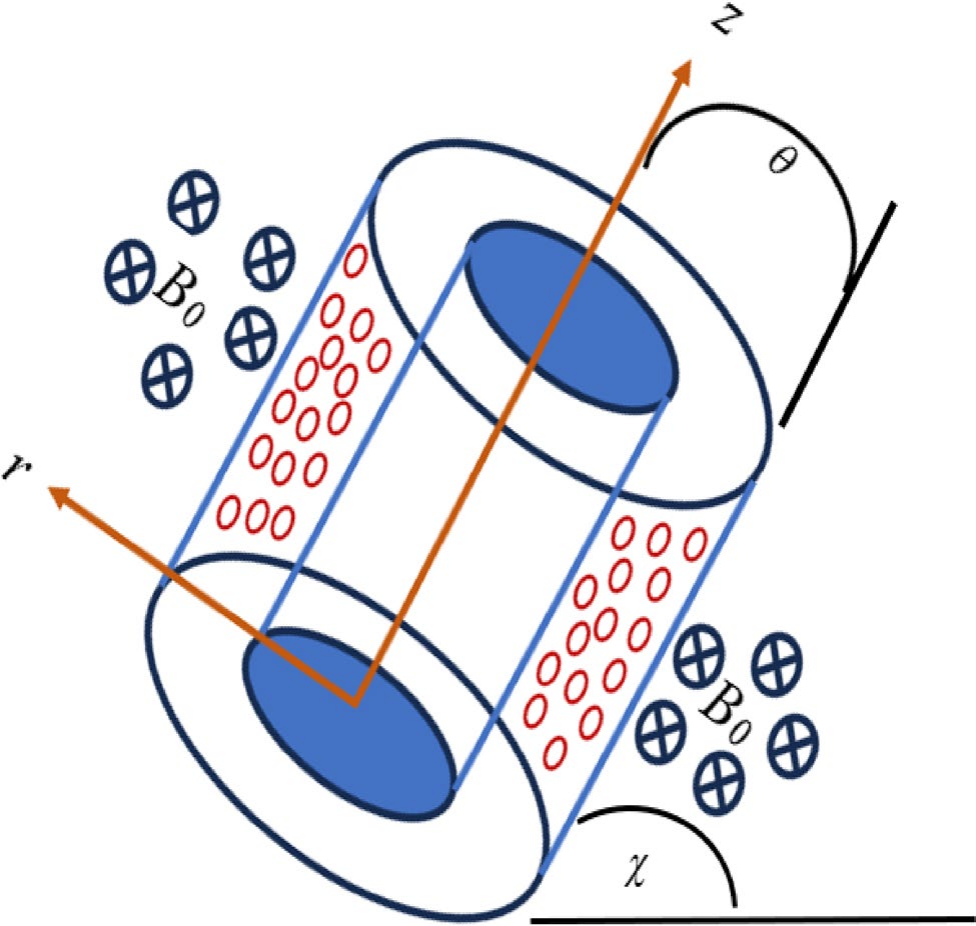
Geometrical configuration of the model.

A fluid-saturated inclined porous annular cavity formed by two concentric cylinders with an internal radius $r_{1}$ and outer radius $r_{2}$ is considered. The innermost and external cylinders are kept at stable but distinct temperatures $T_{c}$ and $T_{h}$. The top and lower annulus surfaces are adiabatic. The $z$ and $r$ axis points towards the height and width of the permeable medium, respectively. Figure 1 denotes the physical representation of the present model, $g$ is the gravity that is parallel to $z$-axis and $\chi$ is the inclination angle. A homogeneous magnetic field $B_{0}$ is exposed perpendicular to both $r$ and $z$ directions. A constant two-dimensional aqueous Fe_3_O_4_ nanofluid flow in an inclined porous annulus is examined by considering radiation, viscous dissipation, and thermophoretic and electrophoretic particle depositions with the following presumptions:

- A porous medium is fluid saturated.
- Each wall is electrically insulated.
- The local thermal equilibrium across the medium is considered.
- In comparison to the applied magnetic field $B_{0}$, the generated magnetic field created by the velocity of an electrically conductive fluid is minimal.
- The porous media is homogenous and isotropic.
- Aside from density fluctuation, fluid characteristics remain constant.

The following mathematical formulas characterize stable, laminar, 2D, MHD dual-diffusive convection in an annulus and are described using the Boussinesq estimation: (Yasuyuki et al.^47^, Wei and Tao^46^).

1 $$\frac{1}{r}\frac{{\partial \left( {r\tilde{u}} \right)}}{\partial r} + \frac{{\partial \tilde{v}}}{\partial z} = 0$$

2 $$\left. \begin{gathered} \tilde{u}\frac{{\partial \tilde{u}}}{\partial r} + \tilde{v}\frac{{\partial \tilde{u}}}{\partial z} = - \frac{1}{{\rho_{nf} }}\frac{\partial p}{{\partial r}} + \frac{{\mu_{nf} }}{{\rho_{nf} }}\left[ {\frac{{\partial^{2} \tilde{u}}}{{\partial r^{2} }} + \frac{1}{r}\frac{{\partial \tilde{u}}}{\partial r} + \frac{{\partial^{2} \tilde{u}}}{{\partial z^{2} }} - \frac{{\tilde{u}}}{{r^{2} }}} \right] - \frac{{\mu_{nf} }}{{\rho_{nf} K}}\tilde{u} \pm \hfill \\ g\beta_{T} \left( {T - T_{c} } \right)\cos \theta \cos \chi + g\beta_{C} \left( {N - N_{c} } \right)\cos \theta \cos \chi - \frac{\sigma }{{\rho_{nf} }}B_{0}^{2} \tilde{u} \hfill \\ \end{gathered} \right\}$$

3 $$\rho_{nf} \left[ {\tilde{u}\frac{{\partial \tilde{v}}}{\partial r} + \tilde{v}\frac{{\partial \tilde{v}}}{\partial z}} \right] = - \frac{\partial p}{{\partial z}} + \mu_{nf} \left[ {\frac{{\partial^{2} \tilde{v}}}{{\partial r^{2} }} + \frac{1}{r}\frac{{\partial \tilde{v}}}{\partial r} + \frac{{\partial^{2} \tilde{v}}}{{\partial z^{2} }} - \frac{{\tilde{v}}}{{r^{2} }}} \right] - \rho_{nf} g\beta_{T} \left( {T - T_{c} } \right)\sin \chi$$

4 $$\left( {\rho c} \right)_{nf} \left[ {\tilde{u}\frac{\partial T}{{\partial r}} + \tilde{v}\frac{\partial T}{{\partial z}}} \right] = k_{nf} \left[ {\frac{{\partial^{2} T}}{{\partial r^{2} }} + \frac{1}{r}\frac{\partial T}{{\partial r}} + \frac{{\partial^{2} T}}{{\partial z^{2} }}} \right] - \frac{1}{r}\frac{\partial }{\partial r}\left( {rq_{r} } \right) + \frac{{\mu_{nf} }}{K}\left( {\tilde{u}^{2} + \tilde{v}^{2} } \right)$$

5 $$\left[ {\tilde{u}\frac{\partial N}{{\partial r}} + \tilde{v}\frac{\partial N}{{\partial z}}} \right] = D_{B} \left[ {\frac{{\partial^{2} N}}{{\partial r^{2} }} + \frac{1}{r}\frac{\partial N}{{\partial r}} + \frac{{\partial^{2} N}}{{\partial z^{2} }}} \right] - \frac{\partial }{\partial r}\left( {u_{t} N} \right) - \frac{\partial }{\partial z}\left( {\left( {v_{t} + v_{g} + v_{e} } \right)N} \right)$$

6 $${\text{The}}\;{\text{Roseland}}\;{\text{radiation}}\;{\text{is}}\;{\text{taken}}\;{\text{as}}\;q_{r} = - \frac{{4\sigma^{*} }}{{3k^{*} }}\frac{{\partial T^{4} }}{\partial r}$$

The thermophoretic velocities $u_{t}$ and $w_{t}$ are written as

7 $$u_{t} = - k_{t} \upsilon_{nf} \frac{1}{T}\frac{\partial T}{{\partial r}}\;{\text{and}}\;v_{t} = - k_{t} \upsilon_{nf} \frac{1}{T}\frac{\partial T}{{\partial z}}$$

$v_{g}$ and $v_{e}$ may be acquired by comparing the Coulomb and gravitational forces to the Stokes drag as

8 $$v_{g} = - \frac{{\rho_{p} d_{p}^{2} C}}{{18\mu_{g} }}g$$

9 $$v_{e} = - \frac{{n_{e} eC}}{{3\pi d_{p} \mu_{g} }}\vec{E}$$

Thermophoretic coefficient defined by

10 $$k_{t} = \frac{{2G_{s} \left( {k_{g} /k_{p} + G_{t} Kn} \right)\left[ {1 + Kn\left( {G_{1} + G_{2} e^{{ - G_{3} /Kn}} } \right)} \right]}}{{\left( {1 + 3G_{m} Kn} \right)\left( {1 + k_{g} /k_{p} + 2G_{t} Kn} \right)}}$$

The boundary conditions are:

11 $$\tilde{u} = 0,\,\,\tilde{v} = 0,\,T = T_{c} ,\,C = C_{c} \,\,at\,\,\,z = 0$$

12 $$\frac{{\partial \tilde{u}}}{\partial z} = 0,\,\,\frac{{\partial \tilde{v}}}{\partial z} = 0,\,\frac{\partial T}{{\partial z}} = \frac{{2qr_{i} }}{{\left( {\rho c} \right)_{p} \left( {r_{o}^{2} - r_{i}^{2} } \right)}},\,\frac{\partial C}{{\partial z}} = C_{0} \,\,at\,\,\,z = L$$

13 $$\tilde{u} = 0,\,\,\tilde{v} = v_{{r_{i} }} ,\,\,\frac{\partial T}{{\partial r}} = 0,\,C = 0\,\,at\,\,\,r = r_{i}$$

14 $$\tilde{u} = 0,\,\,\tilde{v} = v_{{r_{o} }} ,\,\,\frac{\partial T}{{\partial r}} = \frac{q}{k},\,C = C_{h} \,\,at\,r = r_{o}$$

The dimensionless quantities:

15 $$\begin{gathered} \overline{U} = \frac{{\tilde{u}D_{h} }}{{\alpha_{f} }},\overline{V} = \frac{{\tilde{v}D_{h} }}{{\alpha_{f} }},R = \frac{r}{{D_{h} }},Z = \frac{z}{L},A = \frac{L}{{D_{h} }},P = \frac{{pD_{h}^{2} }}{{\rho \alpha_{f}^{2} }},\Pr = \frac{{\upsilon_{f} }}{{\alpha_{f} }}, \hfill \\ \Theta = \frac{{T - T_{c} }}{{T_{h} - T_{c} }},M = B_{0} D_{h} \sqrt {\frac{{\sigma_{f} }}{{\mu_{f} }}} ,Ra_{T} = \frac{{g\beta_{T} \left( {T_{h} - T_{c} } \right)D_{h}^{3} }}{{\alpha_{f} \upsilon_{f} }},\Phi = \frac{N}{{N_{h} - N_{c} }}, \hfill \\ Ra_{S} = \frac{{g\beta_{C} \left( {N_{h} - N_{c} } \right)D_{h}^{3} }}{{\alpha_{f} \upsilon_{f} }},Le = \frac{{\alpha_{f} }}{{D_{B} }} = \frac{Sc}{{\Pr }},b = \frac{{T_{c} }}{{\left( {T_{h} - T_{c} } \right)}},V_{E} = \frac{{v_{e} D_{h} }}{\alpha }, \hfill \\ Da = \frac{K}{{D_{h}^{2} }},\varepsilon = \frac{{\mu_{f} \alpha }}{{\left( {T_{h} - T_{c} } \right)K\left( {\rho c_{p} } \right)_{f} }},Rd = \frac{{16T_{\infty }^{3} \sigma^{*} }}{{3k^{*} \alpha \left( {\rho c_{p} } \right)_{f} }},V_{G} = \frac{{v_{g} D_{h} }}{\alpha }. \hfill \\ \end{gathered}$$

The reduced form of governing equations is:

16 $$\frac{1}{R}\frac{{\partial \left( {RU} \right)}}{\partial R} + \frac{1}{A}\frac{\partial V}{{\partial Z}} = 0$$

17 $$\left. \begin{gathered} U\frac{\partial U}{{\partial R}} + \frac{V}{A}\frac{\partial U}{{\partial Z}} = - \frac{1}{{A_{1} }}\frac{\partial P}{{\partial R}} + \frac{{A_{4} }}{{A_{1} }}\Pr \left[ {\frac{{\partial^{2} U}}{{\partial R^{2} }} + \frac{1}{R}\frac{\partial U}{{\partial R}} + \frac{1}{{A^{2} }}\frac{{\partial^{2} U}}{{\partial Z^{2} }} - \frac{U}{{R^{2} }}} \right] \hfill \\ - \frac{1}{{A_{1} }}M^{2} \Pr U - \frac{{A_{4} }}{{A_{1} }}\frac{\Pr }{{Da}}U \pm \left( {Ra_{T} \Theta + Ra_{N} \Phi } \right)\Pr {\text{Cos}} \theta {\text{Cos}} \chi \hfill \\ \end{gathered} \right\}$$

18 $$U\frac{\partial V}{{\partial R}} + \frac{V}{A}\frac{\partial V}{{\partial Z}} = - \frac{1}{{A_{1} }}\frac{1}{A}\frac{\partial P}{{\partial Z}} + \frac{{A_{4} }}{{A_{1} }}\Pr \left[ {\frac{{\partial^{2} V}}{{\partial R^{2} }} + \frac{1}{R}\frac{\partial V}{{\partial R}} + \frac{1}{{A^{2} }}\frac{{\partial^{2} V}}{{\partial Z^{2} }} - \frac{V}{{R^{2} }}} \right] - Ra_{T} \Pr \Theta {\text{Sin}} \chi$$

19 $$U\frac{\partial \Theta }{{\partial R}} + \frac{V}{A}\frac{\partial \Theta }{{\partial Z}} = \frac{{A_{3} }}{{A_{2} }}\left[ {\frac{{\partial^{2} \Theta }}{{\partial R^{2} }} + \frac{1}{R}\frac{\partial \Theta }{{\partial R}} + \frac{1}{{A^{2} }}\frac{{\partial^{2} \Theta }}{{\partial Z^{2} }}} \right] + \frac{1}{{A_{2} }}Rd\left( {\frac{1}{R}\frac{\partial \Theta }{{\partial R}} + \frac{{\partial^{2} \Theta }}{{\partial R^{2} }}} \right) + \varepsilon \frac{{A_{4} }}{{A_{2} }}\left( {U^{2} + V^{2} } \right)$$

20 $$\left. \begin{gathered} \left[ {U\frac{\partial \Phi }{{\partial R}} + \frac{V}{A}\frac{\partial \Phi }{{\partial Z}}} \right] = \frac{\Pr }{{Sc}}\left[ {\frac{{\partial^{2} \Phi }}{{\partial R^{2} }} + \frac{1}{R}\frac{\partial \Phi }{{\partial R}} + \frac{1}{{A^{2} }}\frac{{\partial^{2} \Phi }}{{\partial Z^{2} }}} \right] + \frac{1}{A}\left( {V_{E} + V_{G} } \right)\frac{\partial \Phi }{{\partial Z}} + \hfill \\ k_{t} \frac{{A_{4} }}{{A_{1} }}\Pr \left( {\Theta + b} \right)^{ - 2} \left( \begin{gathered} \left[ {\left( {\frac{\partial \Theta }{{\partial R}}\frac{\partial \Phi }{{\partial R}} + \Phi \frac{{\partial^{2} \Theta }}{{\partial R^{2} }}} \right)\left( {\Theta + b} \right) - \Phi \left( {\frac{\partial \Theta }{{\partial R}}} \right)^{2} } \right] + \hfill \\ \frac{1}{{A^{2} }}\left[ {\left( {\frac{\partial \Theta }{{\partial Z}}\frac{\partial \Phi }{{\partial Z}} + \Phi \frac{{\partial^{2} \Theta }}{{\partial Z^{2} }}} \right)\left( {\Theta + b} \right) - \Phi \left( {\frac{\partial \Theta }{{\partial Z}}} \right)^{2} } \right] \hfill \\ \end{gathered} \right) \hfill \\ \end{gathered} \right\}$$

Where

21 $$A_{1} = \frac{{\rho_{nf} }}{{\rho_{f} }},A_{2} = \frac{{\left( {\rho c_{p} } \right)_{nf} }}{{\left( {\rho c_{p} } \right)_{f} }},A_{3} = \frac{{k_{nf} }}{{k_{f} }},A_{4} = \frac{{\mu_{nf} }}{{\mu_{f} }}.$$

Thermophysical characterization of aqueous—Fe_3_O_4_ nanofluid is (Chamkha et al.^48^):

22 $$\left. \begin{gathered} \mu_{nf} = \frac{{\mu_{f} }}{{\left( {1 - \varphi } \right)^{2.5} }},\,\rho_{nf} = \left( {1 - \varphi } \right)\rho_{f} + \varphi \rho_{s} , \hfill \\ \left( {\rho c_{p} } \right)_{nf} = \left( {1 - \varphi } \right)\left( {\rho c_{p} } \right)_{f} + \varphi \left( {\rho c_{p} } \right)_{s} \hfill \\ \frac{{k_{nf} }}{{k_{f} }} = \frac{{\left( {k_{s} + 2k_{f} } \right) - 2\left( {k_{f} - k_{s} } \right)\varphi }}{{\left( {k_{s} + 2k_{f} } \right) + \left( {k_{f} - k_{s} } \right)\varphi }} \hfill \\ \end{gathered} \right\}$$

The dimensionless boundary constraints are:

23 $$U = 0,V = 0,\Theta = 0,\Phi = 0\;{\text{at}}\;Z = 0$$

24 $$\frac{\partial U}{{\partial Z}} = 0,\frac{\partial V}{{\partial Z}} = 0,\frac{\partial \Theta }{{\partial Z}} = \frac{4\delta }{{\delta + 1}}\frac{1}{{{\text{Re}} \Pr }},\Phi = 0\,{\text{at}}\,Z = 1$$

25 $$U = 0,V = AZ,\Theta = 1,\Phi = 1\,{\text{at}}\,R = 0$$

26 $$U = 0,V = 0,\frac{\partial \Theta }{{\partial R}} = 0,\frac{\partial \Phi }{{\partial R}} = 0\,{\text{at}}\,R = 1$$

27 $${\text{The}}\;{\text{skin}}\;{\text{friction}}\;{\text{at}}\;{\text{the}}\;{\text{inner}}\;{\text{wall}}\;{\text{is}}\;\tau_{0} = \left. {\frac{\partial U}{{\partial R}}} \right|_{{R = R_{1} }}$$

The parameters of engineering interests are:

28 $$Nu = \left. {\frac{\partial \Theta }{{\partial R}}} \right|_{{R = R_{i} }} \;{\text{and}}\;Nu_{{{\text{avg}}}} = \frac{1}{2\pi L}\int\limits_{0}^{d} {\int\limits_{0}^{L} {Nu} } dZdR$$

29 $$Sh = \left. {\frac{\partial \Phi }{{\partial R}}} \right|_{{R = R_{i} }} \;{\text{and}}\;Sh_{{{\text{avg}}}} = \frac{1}{2\pi L}\int\limits_{0}^{d} {\int\limits_{0}^{L} {Sh} } dZdR$$

## Numerical solution and validation

Equation (16–20) constitutes the coupled nonlinear system of PDEs. The closed-form solution for the current problem is complex and tedious. Hence, these expressions are solved by considering the FDM. The finite difference equations for the system are given below and the properties of the aqueous Fe_3_O_4_ nanofluid are mentioned in Table 1.

$$\left. \begin{gathered} U_{i,j} \frac{{\left( {U_{i + 1,j} - U_{i,j} } \right)}}{\Delta r} + V_{i,j} \frac{{\left( {U_{i,j + 1} - U_{i,j} } \right)}}{A\Delta z} + \frac{\Delta p}{{A_{1} }} \hfill \\ - \frac{{A_{4} \Pr }}{{A_{1} }}\left[ {\frac{{\left( {U_{i + 2,j} - 2U_{i + 1,j} + U_{i,j} } \right)}}{{\left( {\Delta r} \right)^{2} }} + \frac{{\left( {U_{i + 1,j} - U_{i,j} } \right)}}{{i\left( {\Delta r} \right)^{2} }} + \frac{{\left( {U_{i,j + 2} - 2U_{i,j + 1} + U_{i,j} } \right)}}{{\left( {A\Delta z} \right)^{2} }} - \frac{{U_{i,j} }}{{\left( {i\Delta r} \right)^{2} }}} \right] \hfill \\ + \frac{{M^{2} \Pr }}{{A_{1} }}U_{i,j} + \frac{{A_{4} \Pr }}{{A_{1} Da}}U_{i,j} \pm \left( {Ra_{T} \Theta_{i,j} + Ra_{N} \Phi_{i,j} } \right)\Pr {\text{Cos}} \theta {\text{Cos}} \chi = 0 \hfill \\ \end{gathered} \right\}$$

$$\left. \begin{gathered} U_{i,j} \frac{{\left( {V_{i + 1,j} - V_{i,j} } \right)}}{\Delta r} + V_{i,j} \frac{{\left( {V_{i,j + 1} - V_{i,j} } \right)}}{A\Delta z} + \frac{\Delta p}{{AA_{1} }} + Ra_{T} \Theta_{i,j} \Pr {\text{Sin}} \chi - \hfill \\ \frac{{A_{4} \Pr }}{{A_{1} }}\left[ {\frac{{\left( {V_{i + 2,j} - 2V_{i + 1,j} + V_{i,j} } \right)}}{{\left( {\Delta r} \right)^{2} }} + \frac{{\left( {V_{i + 1,j} - V_{i,j} } \right)}}{{i\left( {\Delta r} \right)^{2} }} + \frac{{\left( {V_{i,j + 2} - 2V_{i,j + 1} + V_{i,j} } \right)}}{{\left( {A\Delta z} \right)^{2} }} - \frac{{V_{i,j} }}{{\left( {i\Delta r} \right)^{2} }}} \right] = 0 \hfill \\ \end{gathered} \right\}$$

$$\left. \begin{gathered} U_{i,j} \frac{{\left( {\Theta_{i + 1,j} - \Theta_{i,j} } \right)}}{\Delta r} + V_{i,j} \frac{{\left( {\Theta_{i,j + 1} - \Theta_{i,j} } \right)}}{A\Delta z} - \frac{{A_{3} }}{{A_{2} }}\left[ {\frac{{\left( {\Theta_{i + 2,j} - 2\Theta_{i + 1,j} + \Theta_{i,j} } \right)}}{{\left( {\Delta r} \right)^{2} }} + \frac{{\left( {\Theta_{i + 1,j} - \Theta_{i,j} } \right)}}{{i\left( {\Delta r} \right)^{2} }} + \frac{{\left( {\Theta_{i,j + 2} - 2\Theta_{i,j + 1} + \Theta_{i,j} } \right)}}{{\left( {A\Delta z} \right)^{2} }}} \right] \hfill \\ - \frac{Rd}{{A_{2} }}\left[ {\frac{{\left( {\Theta_{i + 2,j} - 2\Theta_{i + 1,j} + \Theta_{i,j} } \right)}}{{\left( {\Delta r} \right)^{2} }} + \frac{{\left( {\Theta_{i + 1,j} - \Theta_{i,j} } \right)}}{{i\left( {\Delta r} \right)^{2} }}} \right] - \varepsilon \frac{{A_{4} }}{{A_{2} }}\left( {U_{i,j}^{2} + V_{i,j}^{2} } \right) = 0 \hfill \\ \end{gathered} \right\}$$

$$\begin{array}{*{20}l} {U_{i,j} \frac{{\left( {\Phi_{i + 1,j} - \Phi_{i,j} } \right)}}{\Delta r} + V_{i,j} \frac{{\left( {\Phi_{i,j + 1} - \Phi_{i,j} } \right)}}{A\Delta z} - \frac{\Pr }{{Sc}}\left[ {\frac{{\left( {\Phi_{i + 2,j} - 2\Phi_{i + 1,j} + \Phi_{i,j} } \right)}}{{\left( {\Delta r} \right)^{2} }} + \frac{{\left( {\Phi_{i + 1,j} - \Phi_{i,j} } \right)}}{{i\left( {\Delta r} \right)^{2} }} + \frac{{\left( {\Phi_{i,j + 2} - 2\Phi_{i,j + 1} + \Phi_{i,j} } \right)}}{{\left( {A\Delta z} \right)^{2} }}} \right]} \hfill \\ { - \frac{1}{A}\left( {V_{E} + V_{G} } \right)\frac{{\left( {\Phi_{i,j + 1} - \Phi_{i,j} } \right)}}{A\Delta z} - k_{t} \frac{{A_{4} }}{{A_{1} }}\Pr \left( {\Theta_{i,j} + b} \right)^{ - 2} \left( {\left( \begin{gathered} \Phi_{i,j} \frac{{\left( {\Theta_{i + 2,j} - 2\Theta_{i + 1,j} + \Theta_{i,j} } \right)}}{{\left( {\Delta r} \right)^{2} }} \hfill \\ + \frac{{\left( {\Phi_{i + 1,j} - \Phi_{i,j} } \right)\left( {\Theta_{i + 1,j} - \Theta_{i,j} } \right)}}{{\left( {\Delta r} \right)^{2} }} \hfill \\ \end{gathered} \right)\left( {\Theta_{i,j} + b} \right) - \Phi_{i,j} \left( {\frac{{\left( {\Theta_{i + 1,j} - \Theta_{i,j} } \right)}}{\Delta r}} \right)^{2} } \right)} \hfill \\ { - k_{t} \frac{{A_{4} }}{{A_{2} A_{1} }}\Pr \left( {\Theta_{i,j} + b} \right)^{ - 2} \left( {\left( \begin{gathered} \Phi_{i,j} \frac{{\left( {\Theta_{i,j + 2} - 2\Theta_{i,j + 1} + \Theta_{i,j} } \right)}}{{\left( {\Delta z} \right)^{2} }} \hfill \\ + \frac{{\left( {\Phi_{i,j + 1} - \Phi_{i,j} } \right)\left( {\Theta_{i,j + 1} - \Theta_{i,j} } \right)}}{{\left( {\Delta z} \right)^{2} }} \hfill \\ \end{gathered} \right)\left( {\Theta_{i,j} + b} \right) - \Phi_{i,j} \left( {\frac{{\left( {\Theta_{i,j + 1} - \Theta_{i,j} } \right)}}{\Delta z}} \right)^{2} } \right) = 0} \hfill \\ \end{array}$$

**Table 1:** Physical characteristics of the base liquid and nanoparticles^49,50^.

Property	H_2_O	Fe_3_O_4_
$\rho$	997.1	5200
$k$	0.613	6
$c_{p}$	4179	670

**Fig. 2:**
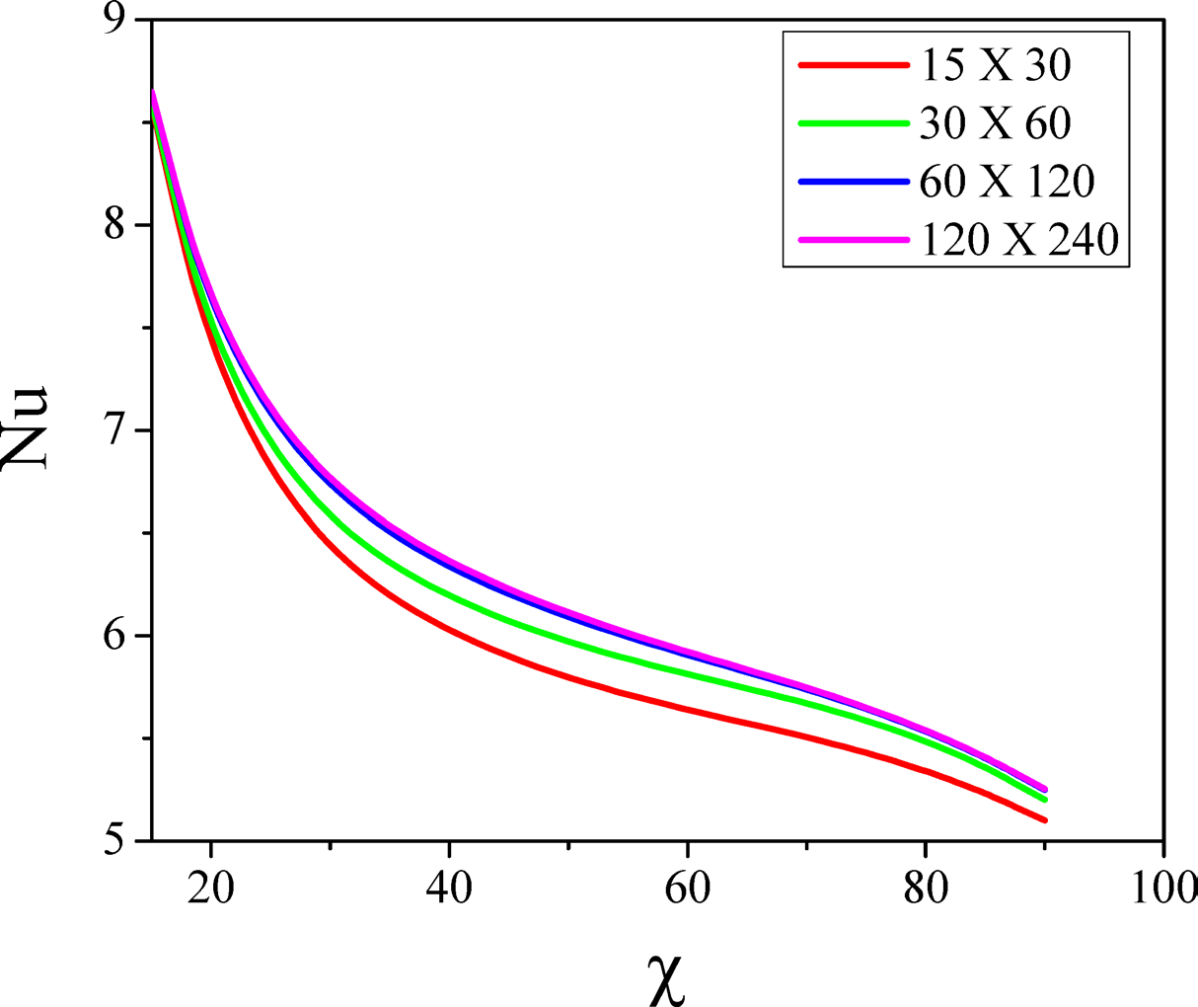
Grid independence study.

**Fig. 3:**
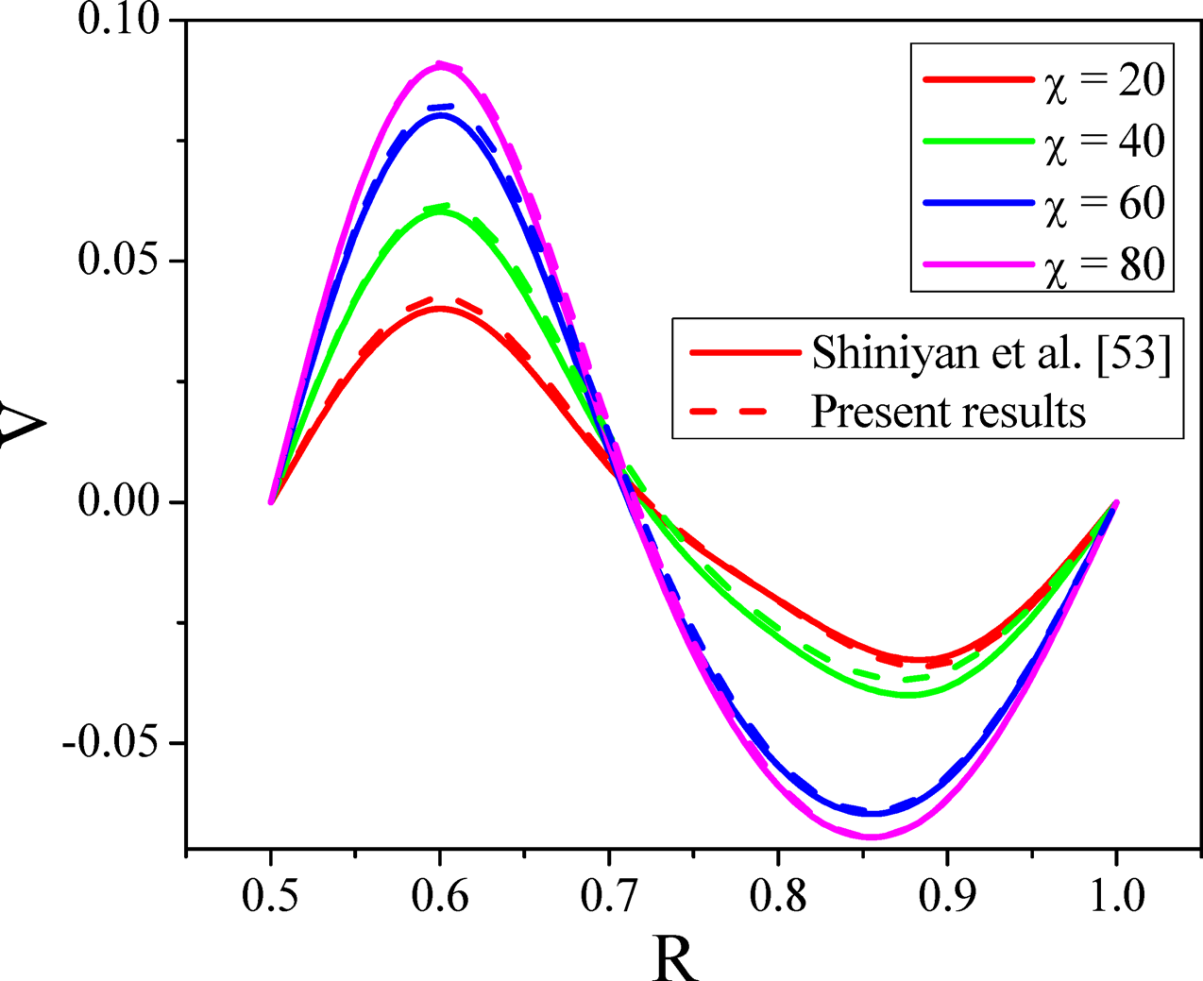
Comparative analysis.

The integration domain is a square with sides $R_{\min } = 0$ to $R_{\max } = 1$ and $Z_{\min } = 0$ to $Z_{\max } = 1$. Here subscript $i$ represents the grid point in $R$, $\left( {i\Delta r} \right)$ and $j$ gives the same in $Z$- direction, $\left( {j\Delta z} \right)$. The grid sizes $30 \times 30,\,60 \times 60,\,120 \times 120$ and $240 \times 240$ (Table 2) are considered to evaluate the Nusselt number and noticed no variations in the Nusselt number profile for the grid sizes $120 \times 120$ and $240 \times 240$(see Fig. 2). Hence, the optimal grid size is considered as $120 \times 120$ for estimation. The resultant tridiagonal system is computed by adopting the Thomas algorithm as stated in^51^. This method is repeated till the desired accuracy is obtained. The desired results are assumed to have been reached when the absolute difference between field variables at two consecutive steps are less than $10^{ - 5}$ at all grids. The procedures are continued for numerous values of parameters, and in the calculations, the $R$ and $Z$ are varied from 0 to 1. The present numerical results are compared with Shiniyan et al.^52^ ‘s existing results under the limiting instances, and an exceptional concurrence is observed between both results (See Fig. 3).

**Table 2:** The grid independence test.

Grid size	Nu	Relative difference
$30 \times 30$	8.5885	0.0054
$60 \times 60$	8.5939	0.0017
$120 \times 120$	8.6109	0.00002
$240 \times 240$	8.61092

## Results and discussion

Double diffusive convection of aqueous—Fe_3_O_4_ nanoliquid flow heat and solute transport in an inclined porous annulus with heat dissipation, thermal radiation, electrophoretic and thermophoretic particle depositions are numerically investigated. The solution for the field variables is obtained through MATLAB software and the graphical illustration is discussed in this section.

**Fig. 4:**
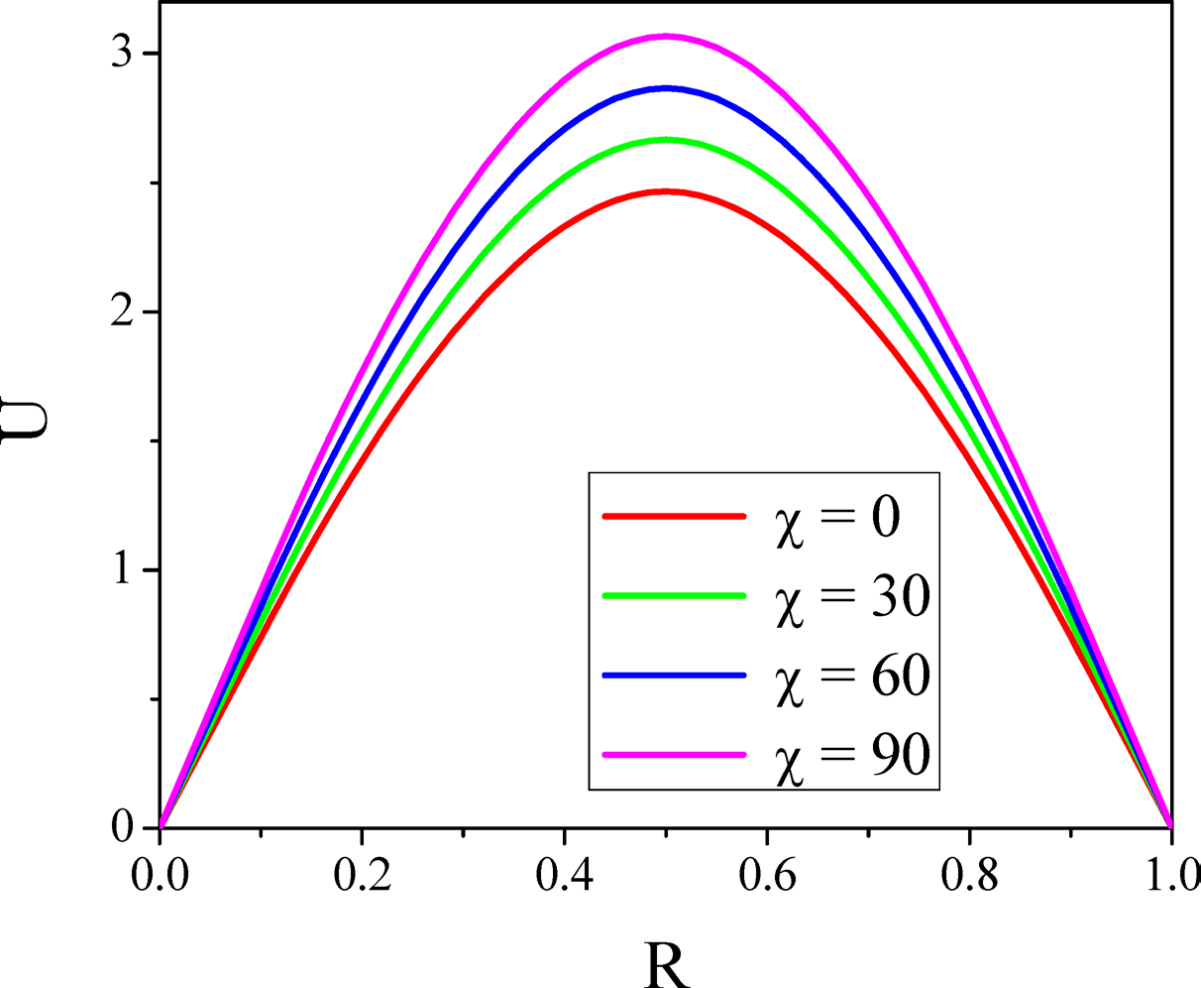
$$U$$ with $$\chi$$.

**Fig. 5:**
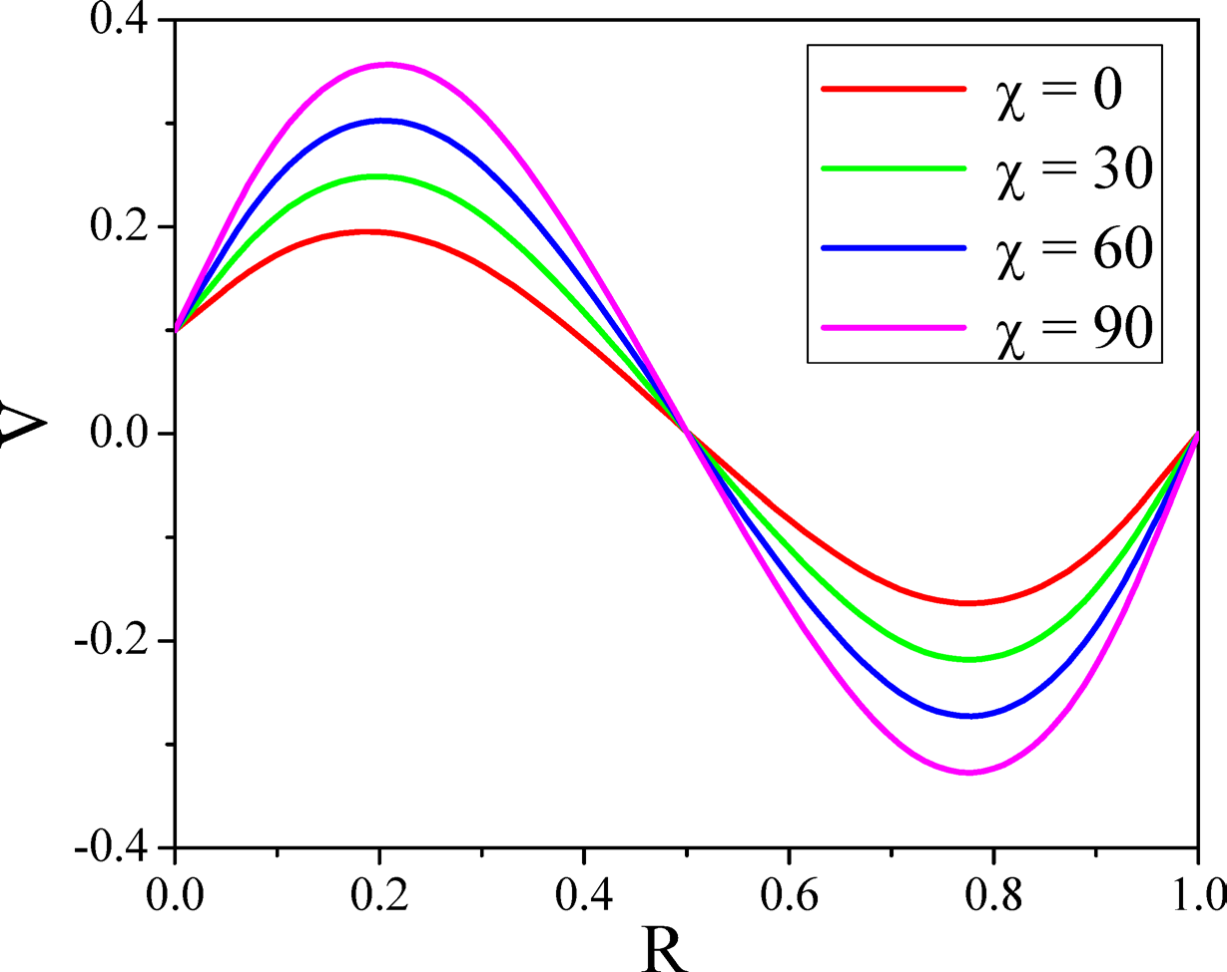
$$V$$ with $$\chi$$.

**Fig. 6:**
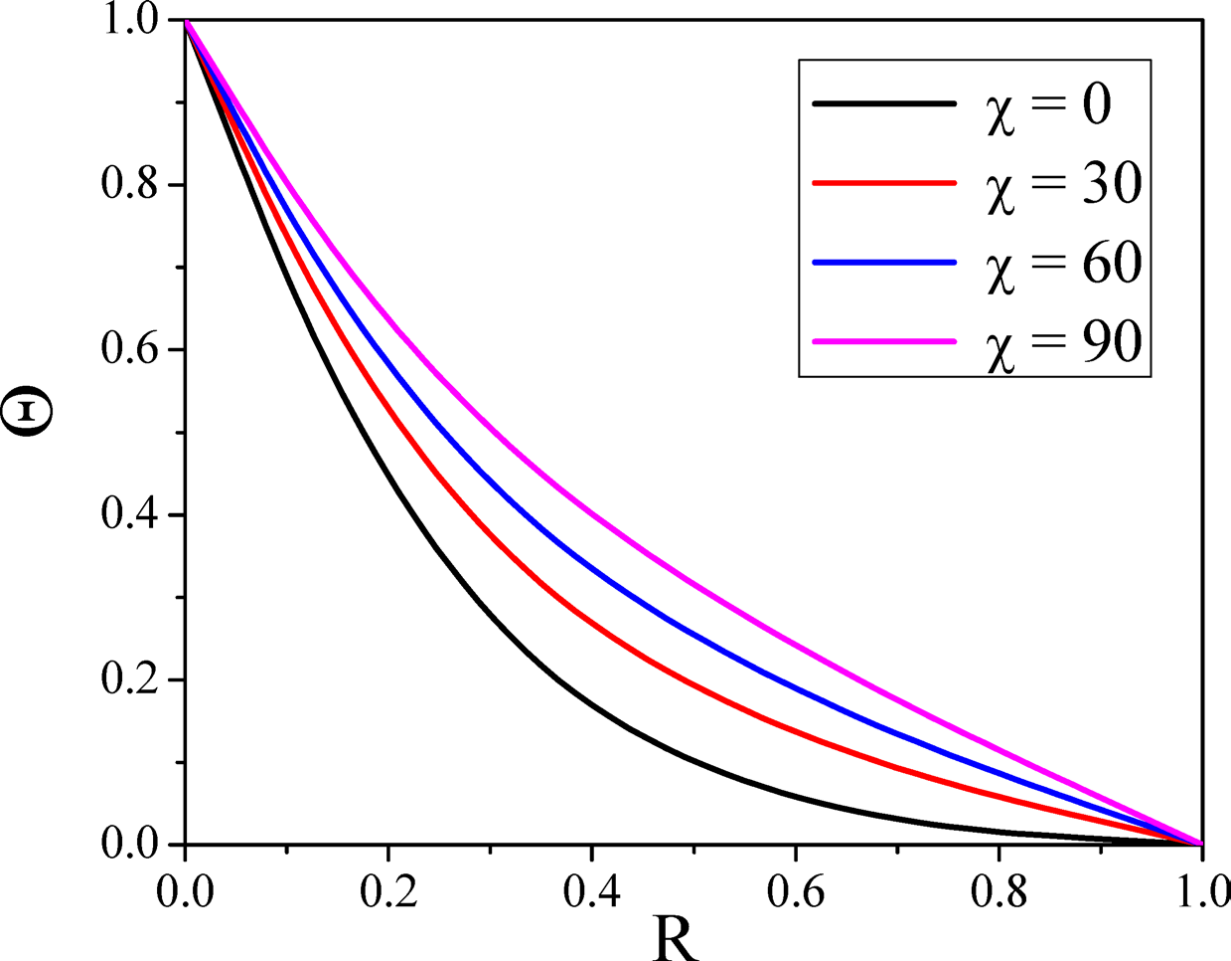
$$\Theta$$ with $$\chi$$.

Figures 4 and 5 illustrate the way $\chi$ affects both axial and radial velocities. Since the convective buoyant force increases with an enhancement in the $\chi$, increasing the $\chi$ increases the fluid flow speed. The buoyancy force causes the higher shear velocity to shift upward when the annulus is laid down, reaching its maximum temperature. For a normal incidence of inclination $\left( {0 \le \chi \le 30^{0} } \right)$, the hydrostatic pressure plays a major role in the axial velocity. Furthermore, the velocity distribution’s maximum is moved upward and enlarged as there is no more angular symmetry in the annulus. As annuli are lowered further $\left( {\chi \ge 30^{0} } \right)$, the secondary flow rate enhances with the percentage of hydrostatic pressure; however, the effect of mixed convective force on the velocity decreases. This causes the greatest axial velocity to go downward when the annulus is horizontal $\left( {\chi \ge 60^{0} } \right)$. The symmetrical shape results in the creation of two vortexes in the secondary flow field, which intensify as the annulus is set down. The impact of the inclination angle parameter on a temperature is seen in Fig. 6. The fluid’s temperature in the porous inclined annulus is raised by the inclination parameter. Rotation increases the fluid particles’ mobility, which transforms the fluid’s kinetic energy into heat energy and raises the temperature.

**Fig. 7:**
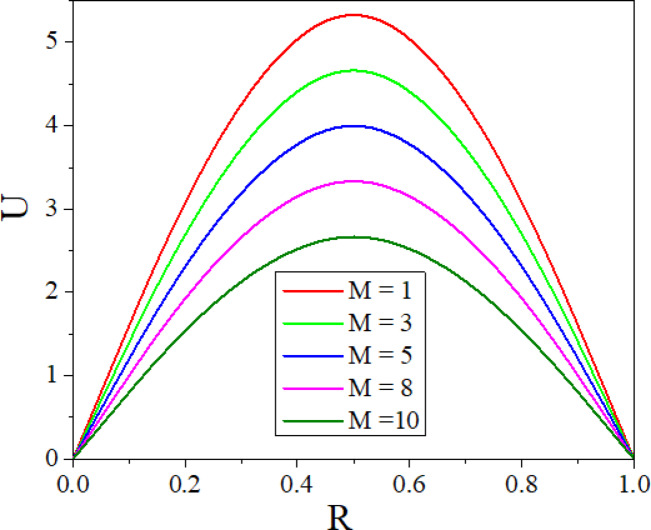
$$U$$ with $$M$$.

**Fig. 8:**
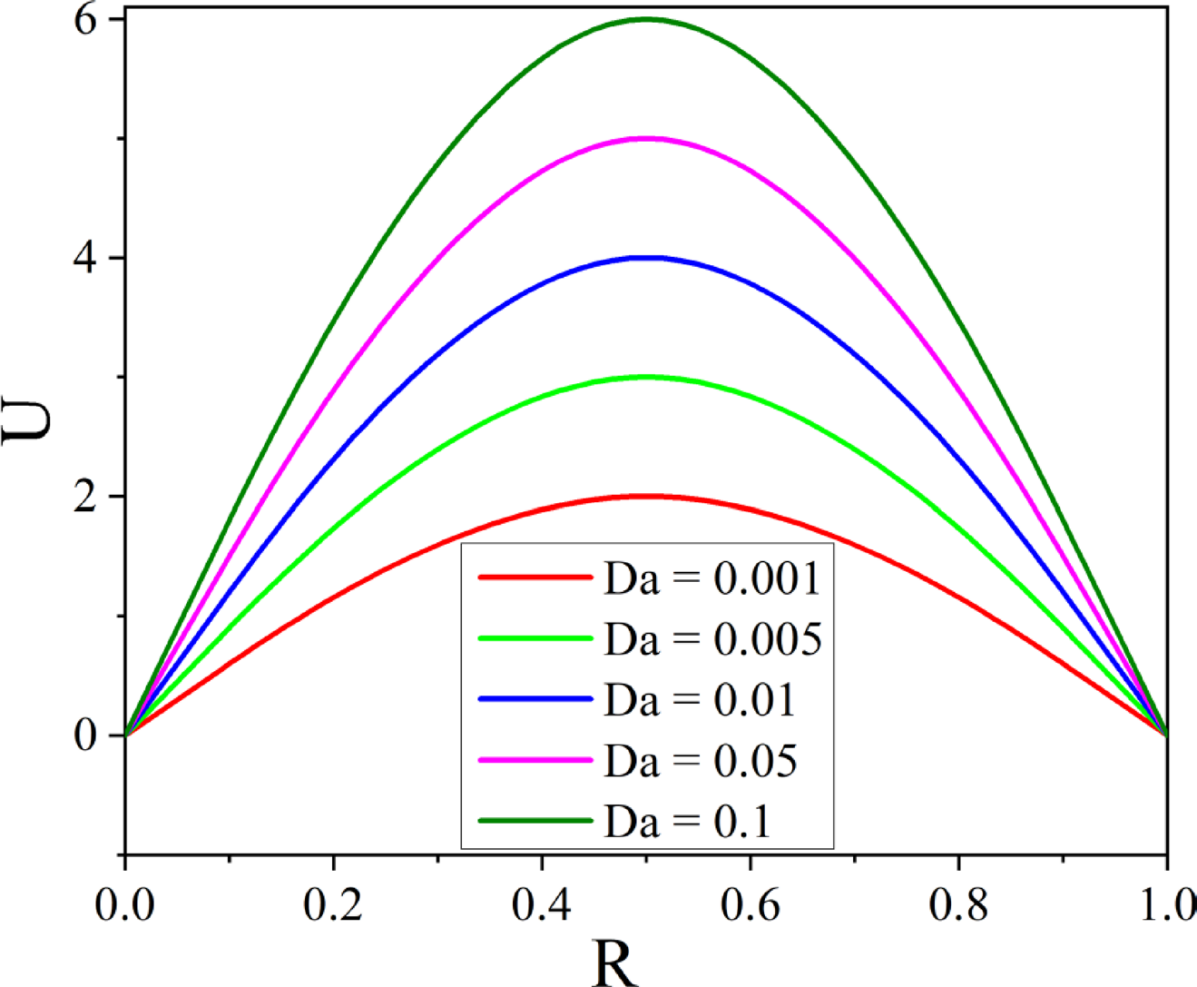
$$U$$ with $$Da$$.

**Fig. 9:**
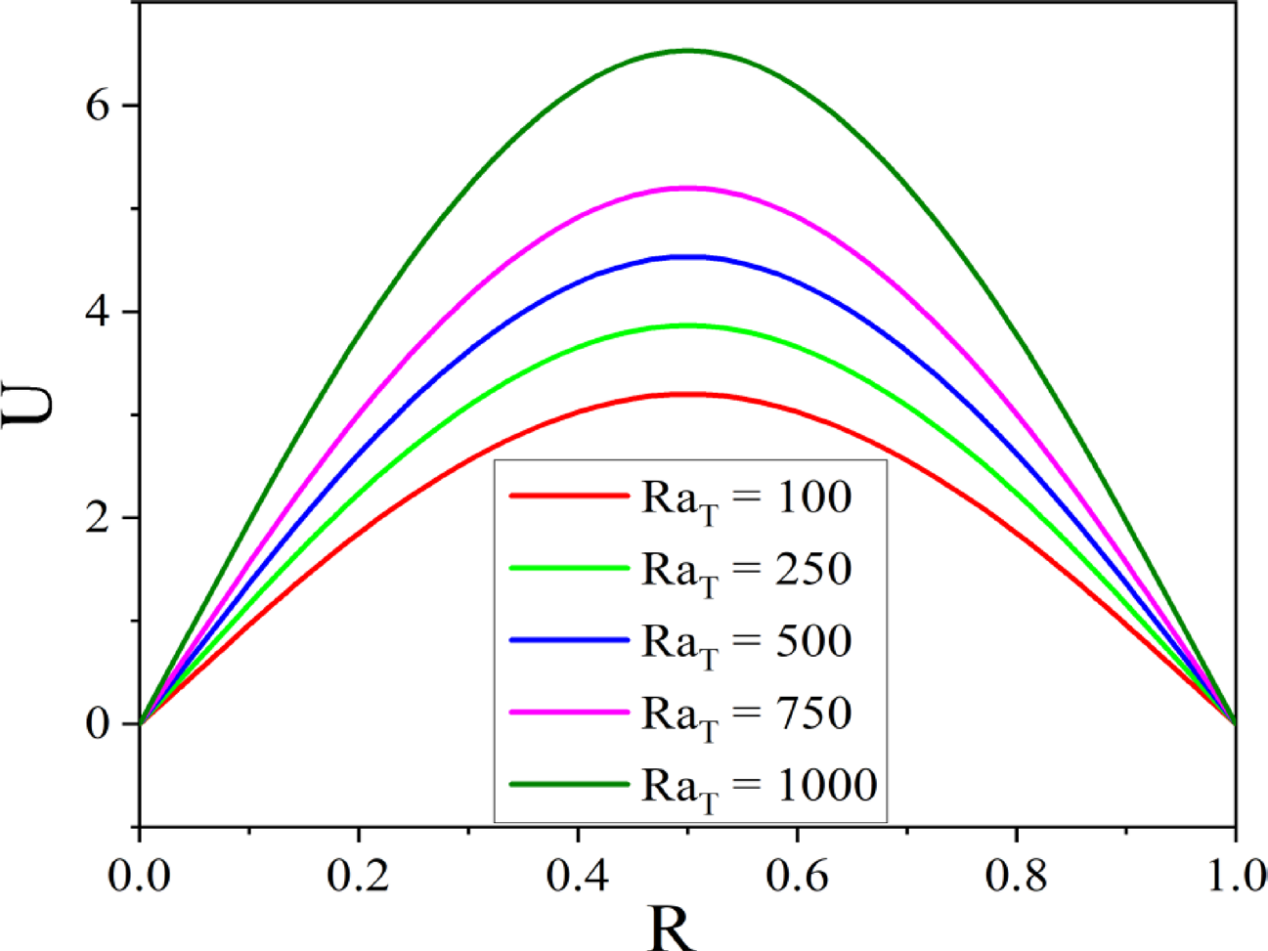
$$U$$ with $$Ra_{T}$$.

**Fig. 10:**
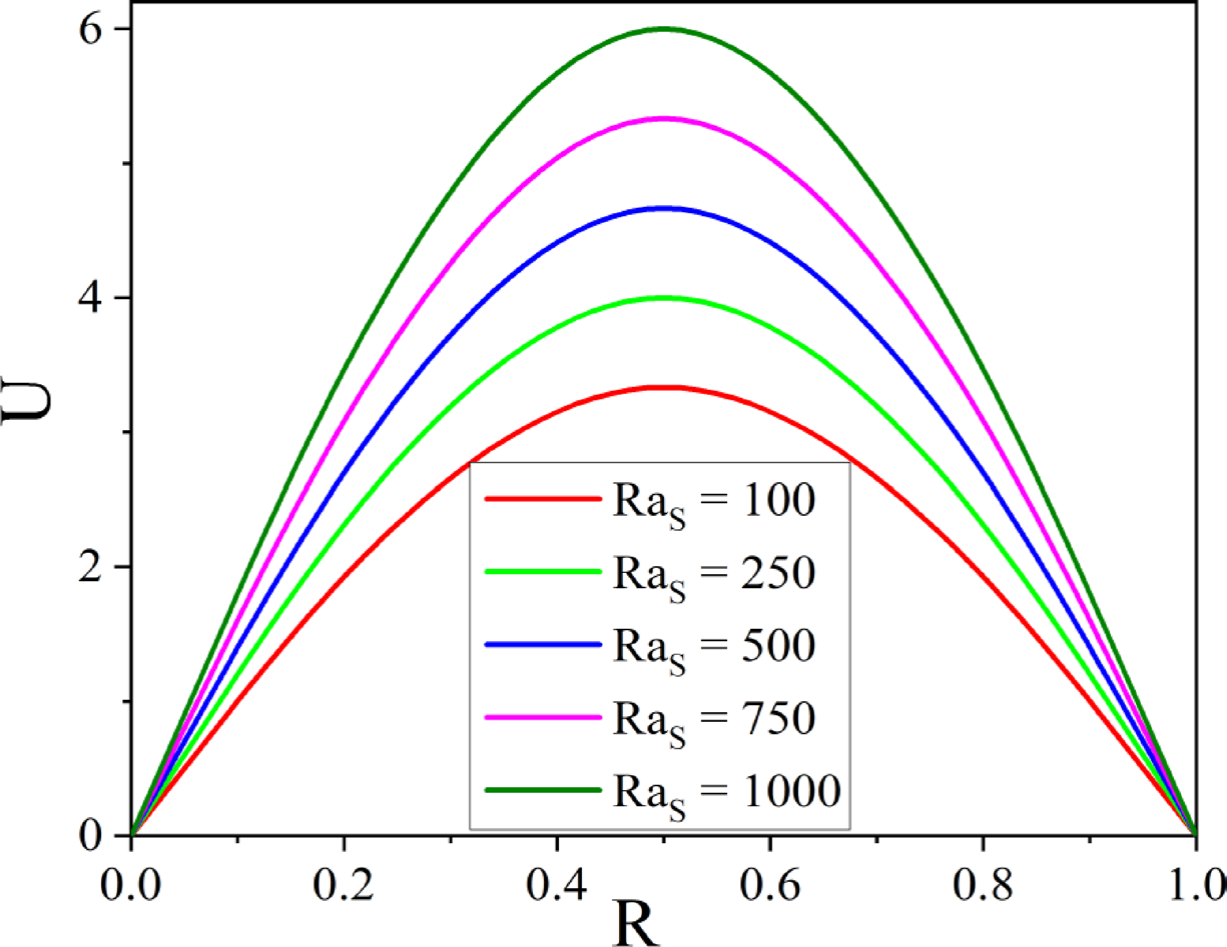
$$U$$ with $$Ra_{S}$$.

The effect of $M$ on velocity is shown in Fig. 7, where the presence of Lorentz force causes the velocity in the annulus to reduce as $M$ grows. When a conductive fluid is applied to a magnetic field, the Lorentz force is produced. By acting in the opposite direction of the flow, this force reduces the fluid velocity and causes flow deceleration. Figure 8 illustrates the way the Darcy number affects fluid flow. As the values of Darcy number enhance the fluid velocity augments in the annulus. A higher Darcy number indicates more permeability of the porous media, allowing the fluid to flow more easily. As a result of the lower resistance, the velocity profile increases, allowing nanoparticles to distribute more uniformly throughout the cross-section. Higher permeability improves convective heat transfer, resulting in a stronger velocity gradient near the boundary. Figures 9 and 10 illustrate the effect of thermal and solutal *Ra* on nanofluid velocity. Both thermal and solutal Rayleigh numbers have a major impact on nanofluid velocity, with greater values of either or both numbers enhancing nanoparticle mobility. The impacts are most noticeable when both values are high, resulting in increased convection and more dynamic nanoparticle motion.

**Fig. 11:**
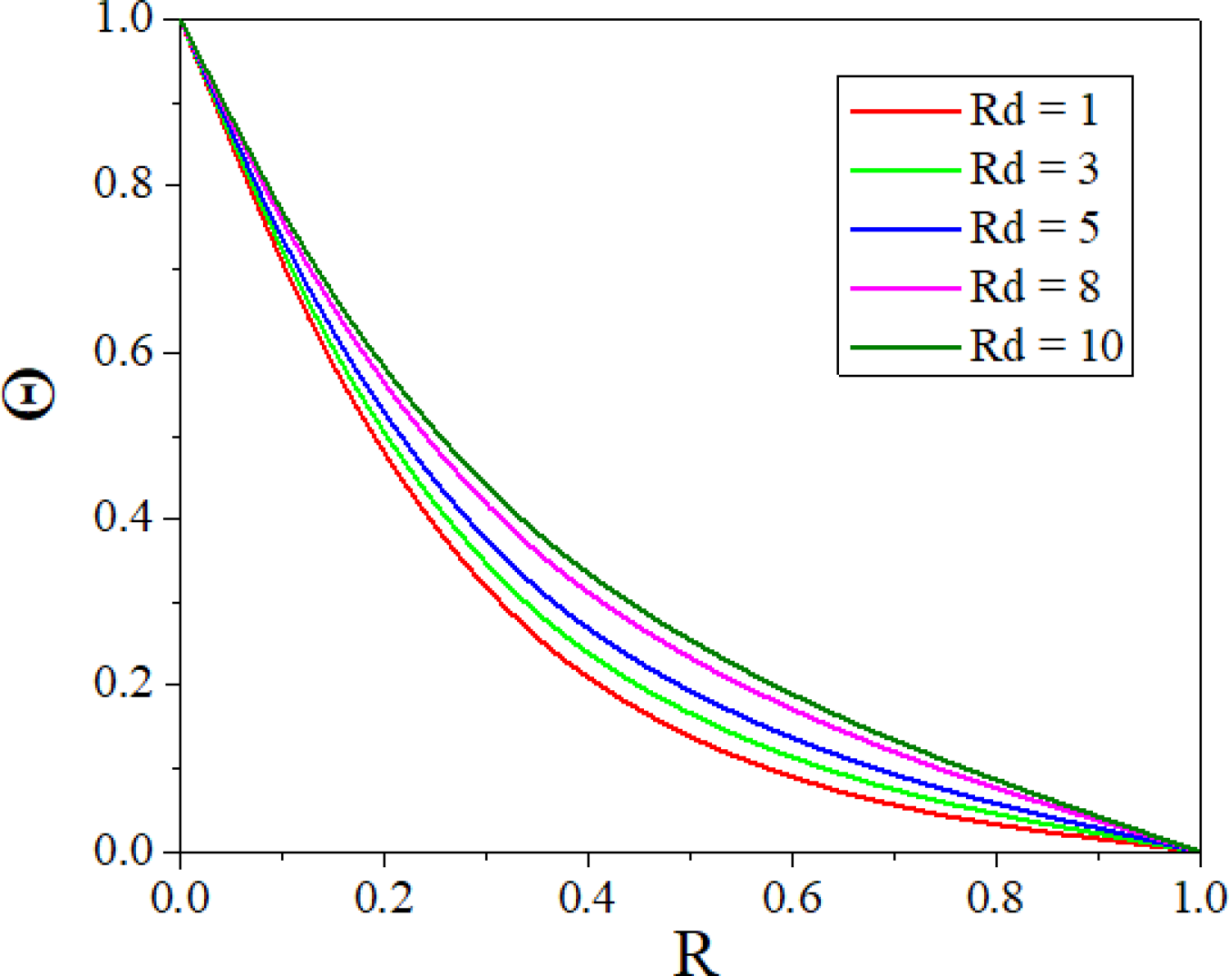
$$\Theta$$ with $$Rd$$.

**Fig. 12:**
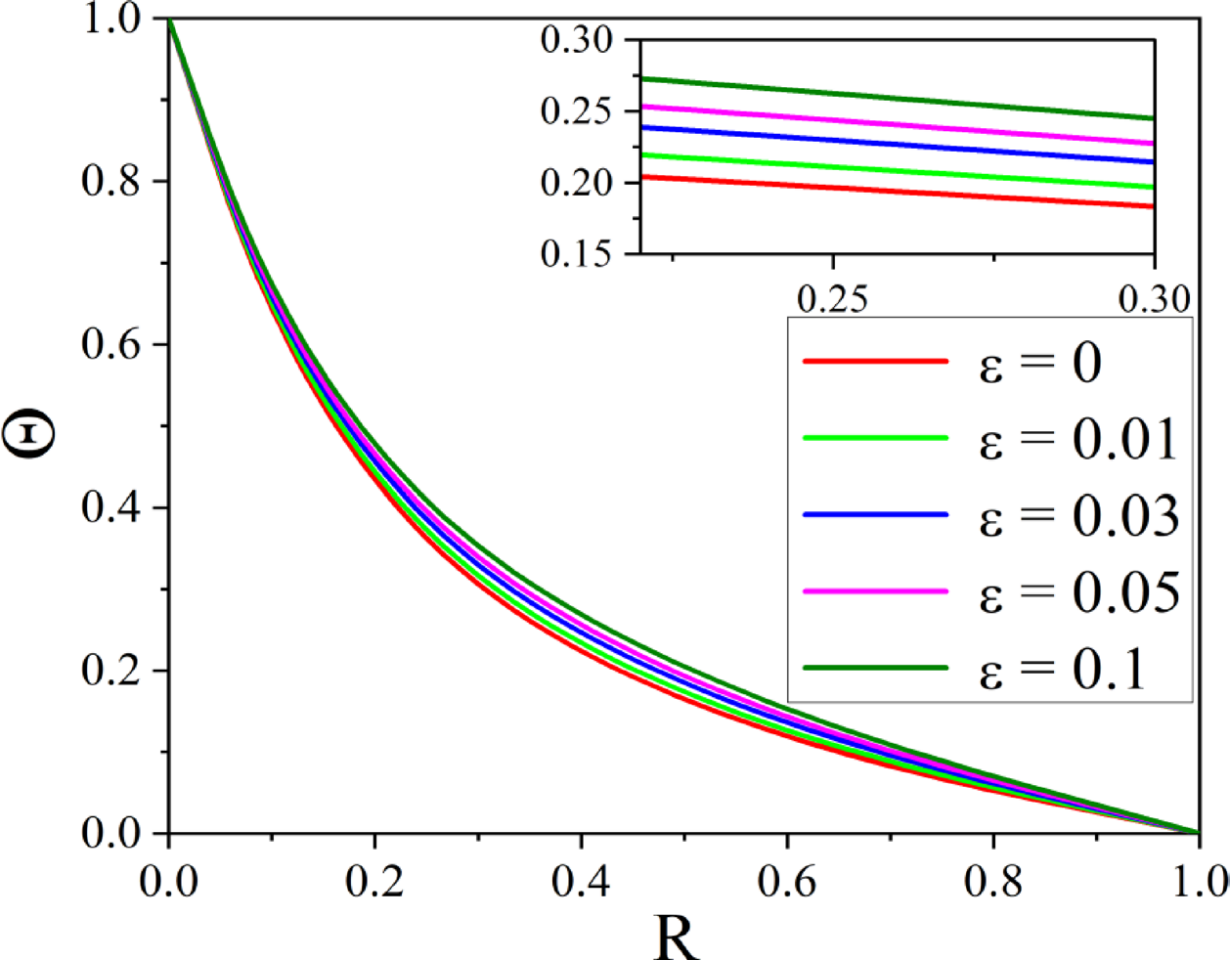
$$\Theta$$ with $$\varepsilon$$.

**Fig. 13:**
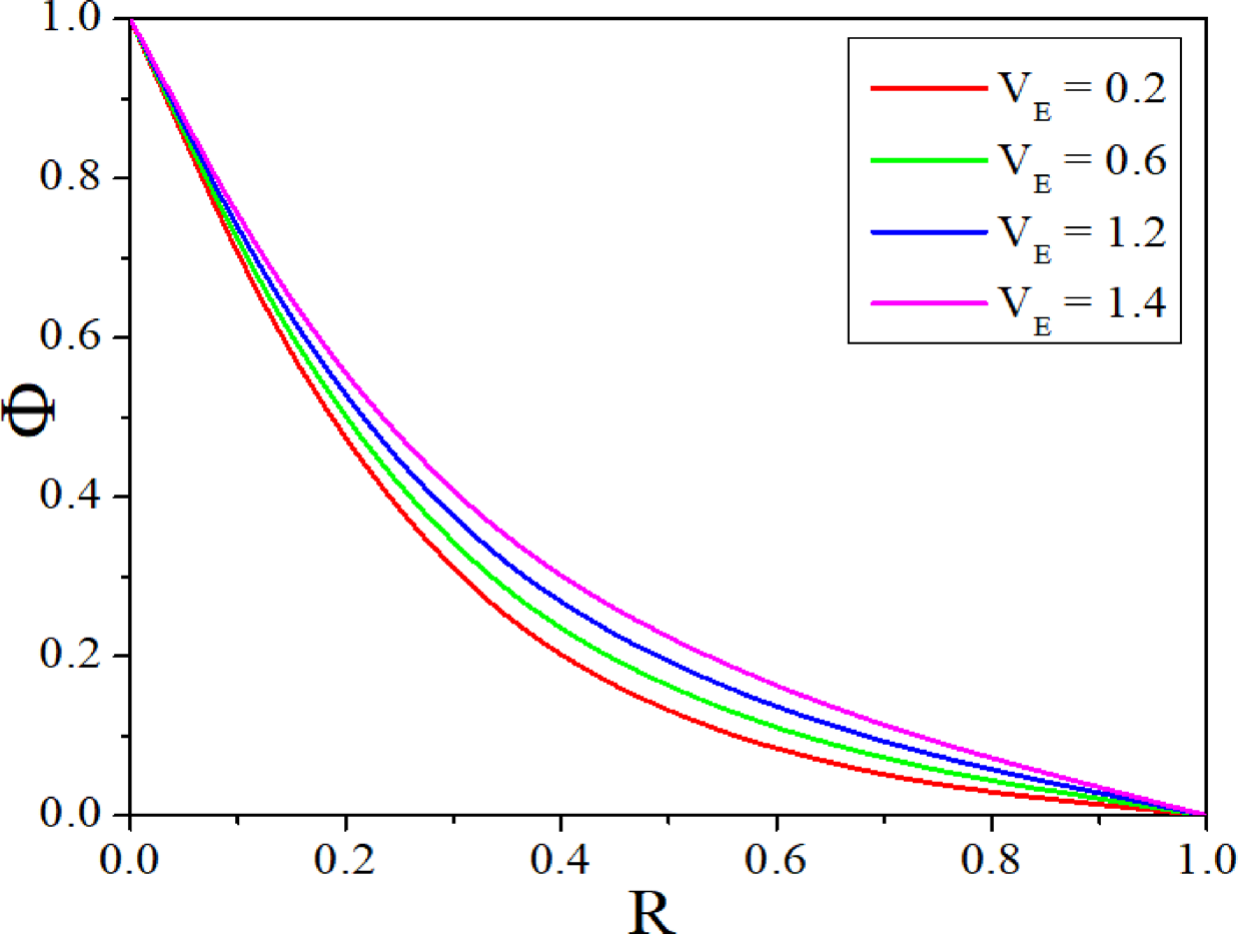
$$\Phi$$ with $$V_{E}$$.

**Fig. 14:**
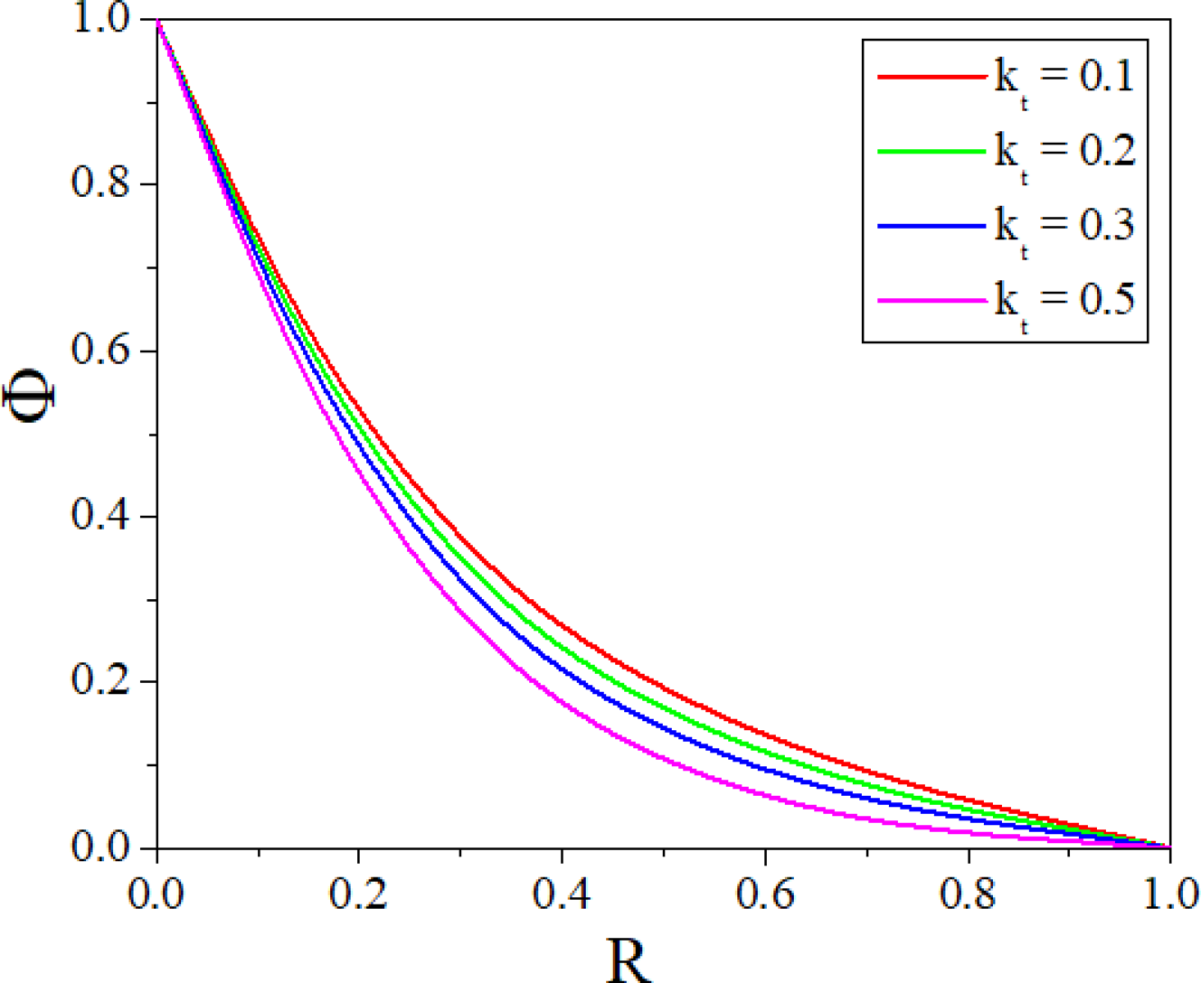
$$\Phi$$ with $$k_{t}$$.

The effect of *Rd* on temperature distribution in a porous annulus can be seen in Fig. 11. As $Rd$ increases the temperature distribution in the annulus increases. Higher values of $Rd$ increases homogeneous temperature distribution and thins the thermal boundary layer, resulting in higher heat transmission. Low $Rd$ causes irregular temperature distribution, sharp gradients around heat sources, and a thick boundary layer. Adjusting $Rd$ can improve thermal efficiency and homogeneity in nanofluid-based heating systems. Figure 12 represents the $\varepsilon$ influence on the temperature profile. As the $\varepsilon$ increases, more mechanical energy is transformed into heat due to internal friction, particularly in the confined, porous structure of the inclined annulus. This increased heat raises the temperature of the nanofluid, particularly near the walls, resulting in a greater total temperature across the annulus. Figures 13 and 14 represent the effect of EPD and TPD on the concentration of the nanofluid. As the values of the EPD parameter enhances the concentration of the nanofluid enhances whereas the concentration diminishes with a rise in the TPD.

**Fig. 15:**
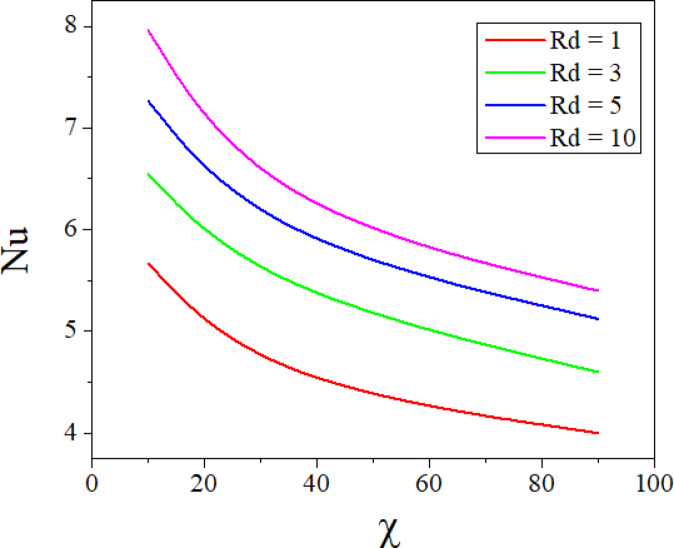
$Rd$ and $\chi$ effect on Nusselt number.

**Fig. 16:**
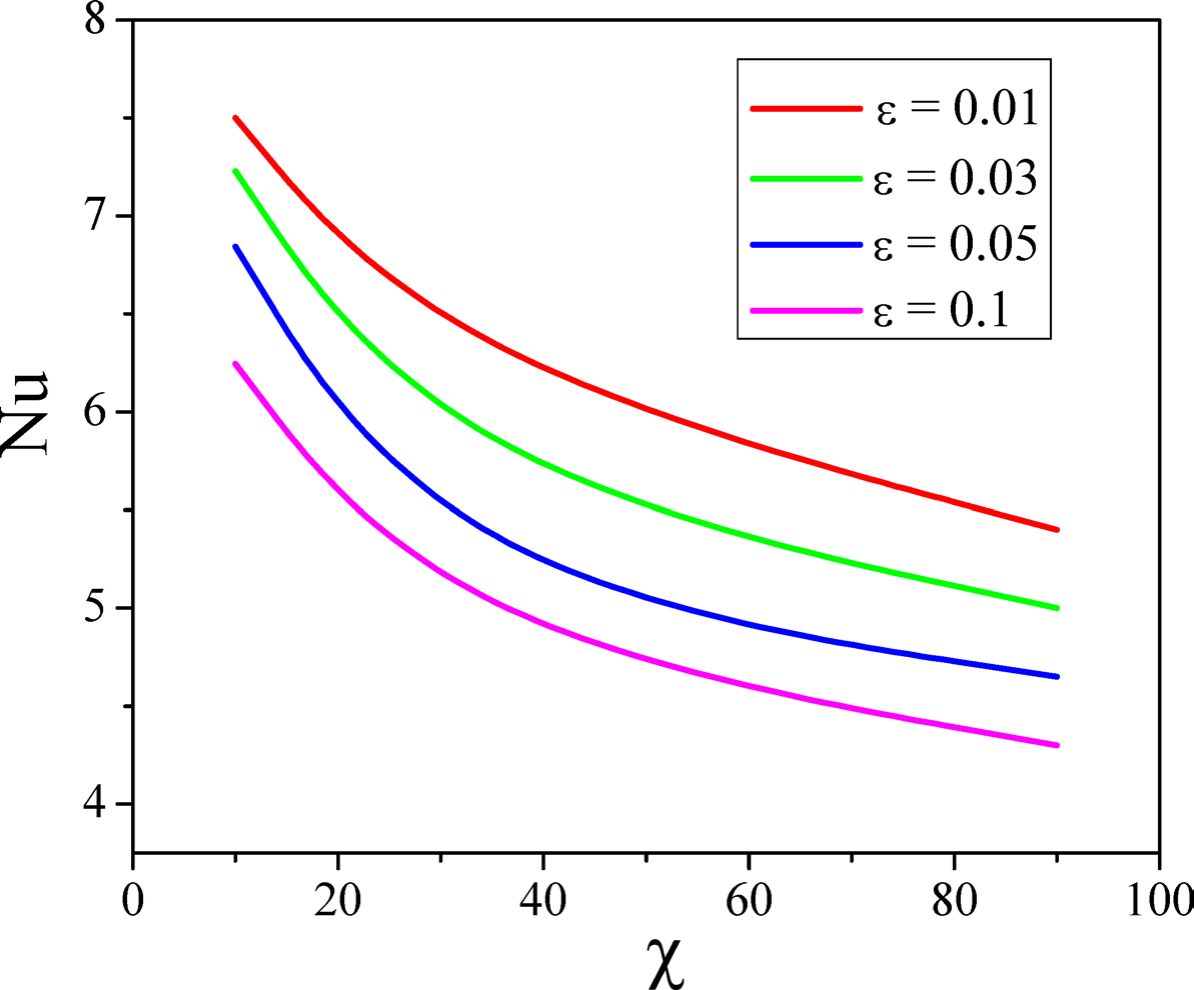
$\varepsilon$ and $\chi$ effect on Nusselt number.

**Fig. 17:**
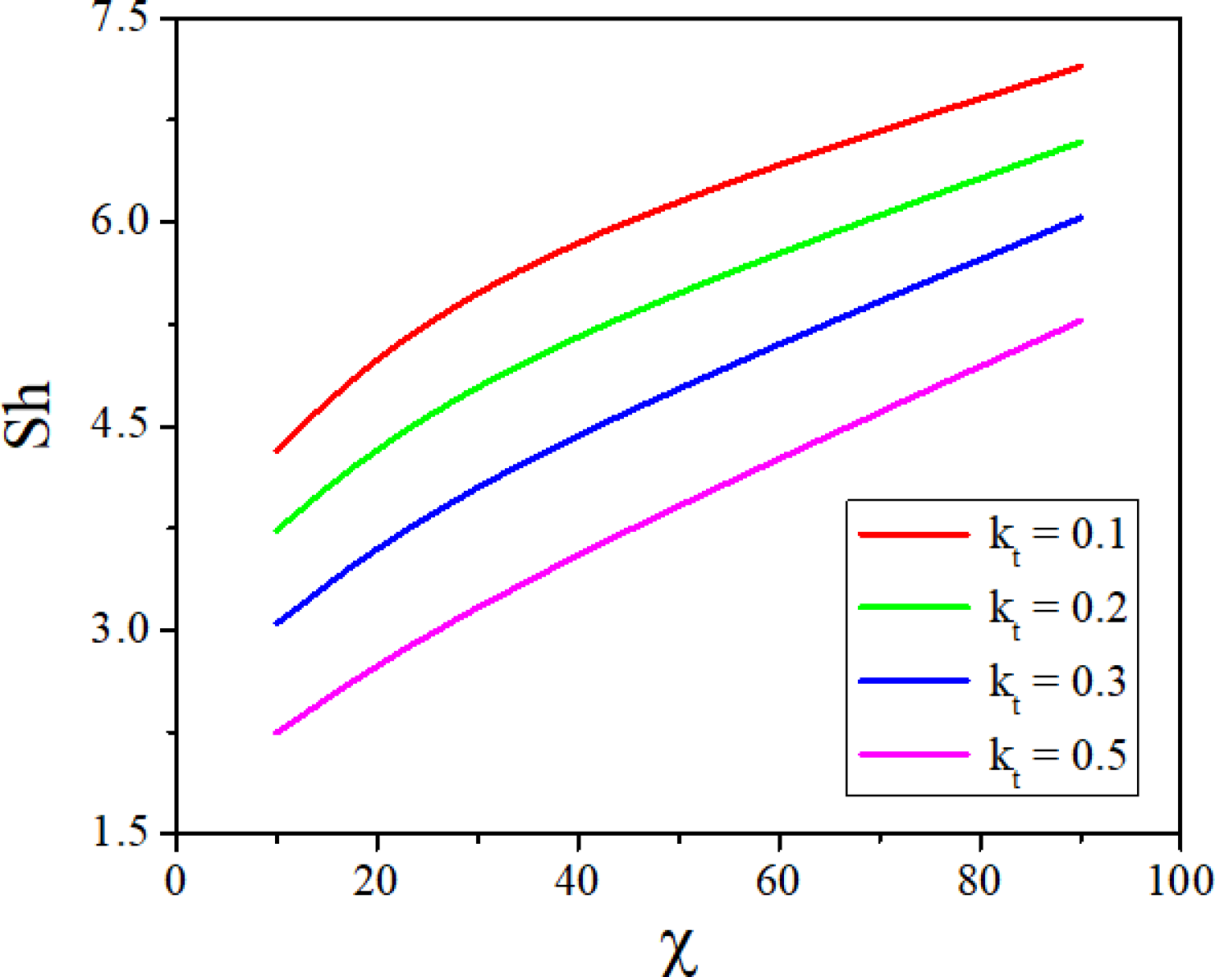
$k_{t}$ and $\chi$ effect on Nusselt number.

**Fig. 18:**
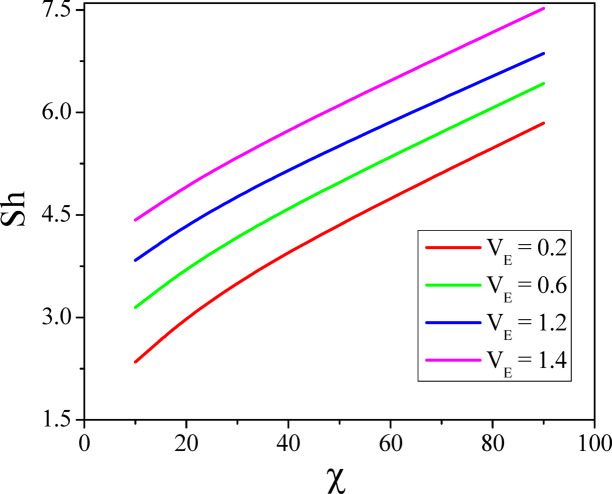
$V_{E}$ and $\chi$ effect on Nusselt number.

The combined effect of inclination angle and the radiation parameter on heat transport rate is analysed in Fig. 15. Increase in the values of $Rd$ increases the heat transfer rate of the nanofluid. Whereas the increased values of $\varepsilon$ diminish the heat transport rate and the same can be seen from Fig. 16. Figures 17 and 18 represent the combined effect of inclination parameter and thermophoretic and electrophoretic parameter effect on mass transfer rate. Higher values of $k_{t}$ enhances the Sherwood number, whereas the opposite trend can be noticed for the greater electrophoretic parameter values.

## Conclusions

The combined influence of EPD and TPD on thermal and solutal transfer of aqueous – Fe_3_O_4_ nanofluid flow in a porous inclined annular region is examined with the effect of an applied magnetic field, radiation, heat dissipation, and double-diffusive convection. The mathematical modelling of the problem resulted in a highly nonlinear coupled system of equations, and these equations are solved by adopting the finite difference method. The results are illustrated and depicted graphically using MATLAB software. The key conclusion of the present problem is as follows.

- For the normal incidence of tilt $\left( {0 \le \chi \le 30^{0} } \right)$, the axial velocity is greatly influenced by the hydrostatic pressure. When the annulus is horizontal $\left( {\chi \ge 60^{0} } \right)$, the maximum axial velocity moves downward in this scenario.
- The Lorentz force is created when a magnetic field is subjected to a conductive fluid which produces a restriction to the fluid flow, hence the velocity reduces in the region.
- Higher permeability improves convective heat transfer, which results in a stronger velocity gradient near the boundary.
- Both thermal and solutal Rayleigh numbers have a major impact on nanofluid velocity, with greater values of either or both numbers nanoparticle movement increases.
- Adjusting $Rd$ can improve thermal efficiency and homogeneity in nanofluid-based heating systems.
- As the viscous dissipation parameter increases, more mechanical energy is converted into heat due to internal friction, particularly in the confined, porous structure of the inclined annulus.
- As the values of the electrophoretic particle deposition parameter enhances the concentration of the nanofluid enhances whereas the concentration diminishes with an increase in the thermophoretic particle deposition parameter.
- Increase in the values of radiation parameter increases the heat transfer rate of the nanofluid. Whereas the increased values of viscous dissipation parameter diminish the heat transfer rate.
- Higher values of the thermophoretic parameter improve the Sherwood number, whereas the reverse trend can be noticed for the greater electrophoretic parameter values.

Several intriguing directions for further research are proposed, building on the results of the current study, which includes extension to complex porous geometries, non-equilibrium thermal modeling, nanoparticle dynamics and stability, pressure drop and pumping power analysis, experimental validation and multi-physics coupling, Optimization and control, energy and economic assessment.

## Data Availability

Upon reasonable request, the corresponding author will provide the datasets used and/or analyzed in the current study.

## List of symbols

$$z$$: Axial direction $$\left( m \right)$$
$$T$$: Temperature $$\left( K \right)$$
$\tilde{u}$: Velocity in the direction of $r$ $\left( {m\,s^{ - 1} } \right)$
$\tilde{v}$: Velocity in the direction of $z$ $\left( {m\,s^{ - 1} } \right)$
$$r$$: Radial path $$\left( m \right)$$
$$g$$: Gravitational force $$\left( {m\,s^{ - 2} } \right)$$
$T_{h} ,\,T_{c}$: Inner and external wall temperature $\left( K \right)$
$$N$$: Concentration
$N_{h} ,\,N_{c}$: Concentration at both the walls
$$B_{0}$$: Magnetic field $$\left( {kg\,A^{ - 1} \,s^{ - 2} } \right)$$
$b$: Temperature difference ratio
$$Le$$: Lewis number
$$k$$: Thermal conductivity $$\left( {kg\,K^{ - 1} \,m\,s^{ - 3} } \right)$$
$c_{p}$: Specific heat capacitance $\left( {K^{ - 1} \,m^{2} \,s^{ - 2} } \right)$
$D_{B}$: Brownian diffusivity coefficient $\left( {m^{2} \,s^{ - 1} } \right)$
$$k^{*}$$: Absorption coefficient $$\left( {m^{ - 1} } \right)$$
$Z$: Dimensionless axial direction
$U$: Dimensionless velocity in $R$ path
$V$: Dimensionless velocity in $Z$ path
$$A$$: Aspect ratio
$P$: Non dimensional pressure
$$p$$: Pressure gradient $$\left( {kg\,m^{ - 1} \,s^{ - 2} } \right)$$
$$M$$: Hartmann number
$Ra_{T}$: Thermal Rayleigh number
$$\Pr$$: Prandtl number
$$D_{h}$$: Hydraulic diameter $$\left( m \right)$$
$Ra_{N}$: Solutal Rayleigh number
$$Rd$$: Radiation term
$$Sc$$: Schmidt number
$$v_{g}$$: Sedimentation velocity
$$L$$: Length
$$v_{e}$$: Electrophoretic velocity
$$U_{T} ,\,V_{T}$$: Thermophoretic velocities
$R$: Dimensionless radial path
$$Nu$$: Nusselt number
$$Sh$$: Sherwood number
$$q_{r}$$: Radiation flux $$\left( {kg\,s^{ - 3} } \right)$$
$$k_{t}$$: Thermophoretic factor
K: Permeability of porous media

## Greek letters

$$\rho$$: Density $$\left( {kg\,m^{ - 3} } \right)$$
$$\mu$$: Dynamic viscosity $$\left( {kg\,s^{ - 1} \,m^{ - 1} } \right)$$
$\beta_{C}$: Volumetric concentration expansion coefficient
$$\sigma$$: Electrical conductivity $$\left( {kg^{ - 1} \,s^{3} \,m^{ - 3} \,A^{2} } \right)$$
$\beta_{T}$: Volumetric thermal expansion coefficient $\left( {K^{ - 1} } \right)$
$$\Theta$$: Dimensionless temperature
$$\Phi$$: Dimensionless concentration
$\sigma^{*}$: Stefan Boltzmann constant $\left( {kg\,s^{ - 3} \,K^{ - 4} } \right)$
$$\theta$$: Polar coordinate
$$\chi$$: Inclination angle
$$\upsilon$$: Kinematic viscosity $$\left( {m^{2} \,s^{ - 1} } \right)$$
$\varepsilon$: Viscous dissipation parameter

## References

[1] Nield, D. A. & Simmons, C. T. A brief introduction to convection in porous media. *Transp. Porous Med.* **130**, 237–250 (2018).

[2] Nield, D. A. & Kuznetsov, A. V. The onset of convection in an anisotropic heterogeneous porous medium: A new hydrodynamic boundary condition. *Transp. Porous Med.* **127**, 549–558. 10.1007/s11242-018-1210-3 (2019).

[3] Nield, D. A. & Bejan, A. Heat Transfer Through a Porous Medium. In *Convection in Porous Media* (eds Nield, D. A. & Bejan, A.) (Springer International Publishing, Cham, 2017).

[4] Vafai, K. & Tien, C. L. Boundary and inertia effects on convective mass transfer in porous media. *Int. J. Heat Mass Trans.* **25**, 1183–1190. 10.1016/0017-9310(82)90212-5 (1982).

[5] Vafai, K. & Thiyagaraja, R. Analysis of flow and heat transfer at the interface region of a porous medium. *Int. J. Heat Mass Trans.* **30**, 1391–1405. 10.1016/0017-9310(87)90171-2 (1987).

[6] Vafai, K. & Tien, C. L. Boundary and inertia effects on flow and heat transfer in porous media. *Int. J. Heat Mass Trans.* **24**, 195–203. 10.1016/0017-9310(81)90027-2 (1981).

[7] Rani, H. P. et al. Numerical analysis of hydromagnetic mixed convective flow in an internally heated vertical porous layer using thermal nonequilibrium model. *Heat Trans.* **51**(7), 6249–6273. 10.1002/htj.22590 (2022).

[8] Leela, V. et al. Numerical investigation on effects of induced magnetic field and viscous dissipation on MHD mixed convection in a vertical micro-porous channel using the brinkman-forchheimer extended Darcy model. *Int. J. Ambient Energy* **43**, 6950–6964. 10.1080/01430750.2022.2059006 (2022).

[9] Hossain, M. A. & Rees, D. A. S. Combined heat and mass transfer in natural convection flow from a vertical wavy surface. *Acta Mech.* **136**, 133–141. 10.1007/BF01179253 (1999).

[10] Shilpa, B. & Leela, V. An artificial intelligence model for heat and mass transfer in an inclined cylindrical annulus with heat generation/absorption and chemical reaction. *Int. Commun. Heat Mass Trans.* **147**, 106956. 10.1016/j.icheatmasstransfer.2023.106956 (2023).

[11] Hossain, M. A., Kabir, S. & Rees, D. A. S. Natural convection of fluid with variable viscosity from a heated vertical wavy surface. *Zeitschrift Für Angewandte Mathematik Und Physik ZAMP* **53**, 48–57. 10.1007/s00033-002-8141-z (2002).

[12] Shilpa, B. et al. Soret and dufour effects on MHD double-diffusive mixed convective heat and mass transfer of couple stress fluid in a channel formed by electrically conducting and non-conducting walls. *Waves Random Complex Med.* **13**, 1–22. 10.1080/17455030.2022.2119491 (2022).

[13] Al-Farhany, K. et al. Magnetohydrodynamic double-diffusive mixed convection in a curvilinear cavity filled with nanofluid and containing conducting fins. *Int. Commun. Heat Mass Trans.* **144**, 106802. 10.1016/j.icheatmasstransfer.2023.106802 (2023).

[14] Gnanasekaran, M. & Satheesh, A. Numerical simulation of mhd double-diffusive mixed convection in a closed cavity filled with liquid potassium alloy: Incorporating thermosolutal source. *Case Stud. Therm. Eng.* **52**, 103772. 10.1016/j.csite.2023.103772 (2023).

[15] Mohammadi, M. & Gandjalikhan Nassab, S. A. Double-diffusive convection flow with soret and dufour effects in an irregular geometry in the presence of thermal radiation. *Int. Commun. Heat Mass Trans.* **134**, 106026. 10.1016/j.icheatmasstransfer.2022.106026 (2022).

[16] Dogonchi, A. S. Heat transfer by natural convection of Fe3O4-water nanofluid in an annulus between a wavy circular cylinder and a rhombus. *Int. J. Heat Mass Trans.* **130**, 320–332. 10.1016/j.ijheatmasstransfer.2018.10.086 (2019).

[17] Hosseinzadeh, M. et al. Convective heat transfer and friction factor of aqueous Fe3O4 nanofluid flow under laminar regime. *J. Therm Anal. Calorim* **124**, 827–838. 10.1007/s10973-015-5113-z (2016).

[18] Thirumalaisamy, K., Sivaraj, R. & Subramanyam Reddy, A. Fluid flow and heat transfer analysis of a ternary aqueous Fe3O4 + MWCNT + Cu/H2O magnetic nanofluid in an inclined rectangular porous cavity. *J. Magnet. Magnet. Mater.* **589**, 171503. 10.1016/j.jmmm.2023.171503 (2024).

[19] Shilpa, B. & Leela, V. Galerkin finite element analysis of heat and mass transfer of Jeffrey, Maxwell and Oldroyd-B nanofluids in a Vertical annulus with an induced magnetic field and a non-uniform heat source/sink. *Int. J. Ambient Energy* **44**, 1887–1903. 10.1080/01430750.2023.2196988 (2023).

[20] Suneetha, S., Narayana Naik, R., Srinivasa Babu, K. S. & Babu, M. J. Entropy generation analysis of Carreau-Yasuda hybrid nanofluid flow with Thompson-Troian boundary conditions and Cattaneo-Christov heat flux. *Int. J. Ambient Energy* **46**(1), 2483919 (2025).

[21] Sangapatnam, S., Rajavath, N. N., Kasibhotla, S. S. B. & Jayachandra Babu, M. Impact of shape factors on the flow of Ree-Eyring hybrid nanofluid with Thompson-Troian boundary conditions: an irreversibility analysis. *World J. Eng.*10.1108/WJE-09-2024-0508 (2025).

[22] Gadamsetty Revathi, M., Jayachandra Babu, K. S., Babu, S. & Bapanayya, C. Dynamics of different heat sources and activation energy on the hybrid nanofluid (EG + MgO + MWCNT) flow in a microchannel with thermal radiation: An irreversibility analysis. *Numer. Heat Trans. Part A: Appl.*10.1080/10407782.2024.2316222 (2024).

[23] Revathia, G., Gopinath Veeram, M., Jayachandra Babu, K. S., Babu, S. & Suneel Kumar, A. Darcy-Forchheimer flow of power-law (Ostwald-de Waele type) nanofluid over an inclined plate with thermal radiation and activation energy: an irreversibility analysis. *Int. J. Ambient Energy* **44**(1), 1980–1989 (2023).

[24] Sedki, A. M. Effect of thermal radiation and chemical reaction on MHD mixed convective heat and mass transfer in nanofluid flow due to nonlinear stretching surface through porous medium. *Results Mater.* **16**, 100334. 10.1016/j.rinma.2022.100334 (2022).

[25] Badruddin, I. A. et al. Numerical analysis of convection conduction and radiation using a non-equilibrium model in a square porous cavity. *Int. J. Therm. Sci.* **46**, 20–29. 10.1016/j.ijthermalsci.2006.03.006 (2007).

[26] Fand, M. R. & Brucker, J. Correlation for heat transfer by natural convection from horizontal cylinders that accounts for viscous dissipation. *Int. J. Heat Mass Trans.* **26**, 709–716 (1983).

[27] Fand, M. R., Steinberger, T. E. & Cheng, P. Natural convection heat transfer from a horizontal cylinder embedded in a porous medium. *Int. J. Heat Mass Trans.* **29**, 119–133 (1986).

[28] Saeid, I. & Pop, N. H. Viscous dissipation effects on free convection in a porous cavity. *Int. Commun. Heat Mass Transf.* **31**, 723–732 (2004).

[29] Israel-Cookey, A. & Ogulu, V. B. Omubo-Pepple, Influence of viscous dissipation and radiation on unsteady MHD free-convection flow past an infinite heated vertical plate in a porous medium with time-dependent suction. *Int. J. Heat Mass Transf.* **46**, 2305–2311 (2003).

[30] Badruddin, I. A. et al. Heat transfer in porous cavity under the influence of radiation and viscous dissipation. *Int. Commun. Heat Mass Transf.* **33**, 491–499. 10.1016/j.icheatmasstransfer.2006.01.015 (2006).

[31] Badruddin, I. A. et al. Effect of viscous dissipation and radiation on natural convection in a porous medium embedded within vertical annulus. *Int. J. Therm. Sci.* **46**, 221–227. 10.1016/j.ijthermalsci.2006.05.005 (2007).

[32] Talbot, L., Cheng, R. K., Schefer, R. W. & Willis, D. R. Thermophoresis of particles in a heated boundary layer. *J. Fluid Mech.* **101**, 737–758 (1980).

[33] Batchelor, G. K. & Shen, C. Thermophoretic deposition of particles in gas flowing over cold surfaces. *J. Colloid Interface Sci.* **107**, 21–37 (1985).

[34] Goren, S. L. Thermophoresis of aerosol particles in the laminar boundary layer on a flat plate. *J. Colloid Interface Sci.* **61**, 77–85 (1977).

[35] Tsai, R. & Liang, L. J. Correlation for thermophoretic deposition of aerosol particles onto cold plates. *J. Aerosol Sci.* **32**, 473–487 (2001).

[36] Postelnicu, A. Effects of thermophoresis particle deposition in free convection boundary layer from a horizontal flat plate embedded in a porous medium. *Int. J. Heat Mass Transf.* **50**, 2981–2985 (2007).

[37] Shilpa, B. et al. Exploration of linear and exponential heat source/sink with the significance of thermophoretic particle deposition on ZnO-SAE50 nano lubricant flow past a curved surface. *Case Stud. Therm. Eng.* **61**, 104883. 10.1016/j.csite.2024.104883 (2024).

[38] Shilpa, B. et al. Integrated neural network based simulation of thermo solutal radiative double-diffusive convection of ternary hybrid nanofluid flow in an inclined annulus with thermophoretic particle deposition. *Case Stud. Therm. Eng.* **62**, 105158. 10.1016/j.csite.2024.105158 (2024).

[39] Cooper, D. W., Peters, M. H. & Miller, R. J. Predicted deposition of submicrometer particles due to diffusion and electrostatics in viscous axisymmetric stagnation point flow. *Aerosol Sci. Technol.* **11**, 133–143 (1989).

[40] Turner, J. R., Liguras, D. K. & Fissan, H. J. Clean room applications of particle deposition from stagnation flow: Electrostatic effects. *J. Aerosol. Sci.* **20**, 403–417 (1989).

[41] Hwang, J. & Daily, J. W. Electric field enhanced deposition in flame-synthesized materials manufacturing. *J. Aerosol. Sci.* **26**, 5–18 (1995).

[42] Tsai, R. & Huang, J. S. Combined effects of thermophoresis and electrophoresis on particle deposition onto a vertical flat plate from mixed convection flow through a porous medium. *Chem. Eng. J.* **157**, 52–59 (2010).

[43] Ali, J. Chamkha, I Pop, Effect of thermophoresis particle deposition in free convection boundary layer from a vertical flat plate embedded in a porous medium. *Int. Commun. Heat Mass Transf.* **31**(3), 421–430 (2004).

[44] Gangadhar, K., Kumari, M. A. & Chamkha, A. J. EMHD flow of radiative second-grade nanofluid over a riga plate due to convective heating: Revised Buongiorno’s nanofluid model. *Arab. J. Sci. Eng.* **47**, 8093–8103 (2022).

[45] Parvin, S., Rehena Nasrin, M. A., Alim, N. F. H. & Chamkha, A. J. Thermal conductivity variation on natural convection flow of water–alumina nanofluid in an annulus. *Int. J. Heat Mass Transf.* **55**(19–20), 5268–5274 (2012).

[46] Wei, J. G. & Tao, W. Q. Three-dimensional numerical simulation of natural convection heat transfer in an inclined cylindrical annulus. *J. Therm. Sci.* **5**(3), 175–183 (1996).

[47] Takata, Y., Iwashige, K., Fukuda, K. & Hasegawa, S. Three-dimensional natural convection in an inclined cylindrical annulus. *Int. J. Heat Mass Trans.* **27**(141), 154 (1984).

[48] Chamkha, A. J., Rashad, A. M., El-Zahar, E. R. & El-Mky, H. A. Analytical and numerical investigation of Fe3O4–water nanofluid flow over a moveable plane in a parallel stream with high suction. *Energies* **12**, 198. 10.3390/en12010198 (2019).

[49] Noghrehabadi, A., Behseresht, A., Ghalambaz, M. & Behseresht, J. Natural convection flow of nano fluids over a vertical cone embedded in a non-Darcy porous medium. *J. Thermophys. Heat Transf.* **27**, 334–341 (2013).

[50] Behseresht, A., Noghrehabadi, A. & Ghalambaz, M. Natural convection heat and mass transfer from a vertical cone in porous media filled with nano fluids using the practical ranges of nano fluids thermophysical properties. *Chem. Eng. Res. Des.* **92**, 447–452 (2014).

[51] Carnahan, B., Luther, H. A. & Wilkes, J. O. *Applied Numerical Methods* 44 (John Wiley and Sons, 1969).

[52] Shiniyan, B., Hosseini, R. & Naderan, H. Numerical study on flow and thermal fields of mixed convection in a concentric inclined annulus. *Heat Transf. Eng.* **37**(9), 751–762. 10.1080/01457632.2015.1080557 (2016).

